# A small molecule inhibitor prevents gut bacterial genotoxin production

**DOI:** 10.1038/s41589-022-01147-8

**Published:** 2022-10-17

**Authors:** Matthew R. Volpe, José A. Velilla, Martin Daniel-Ivad, Jenny J. Yao, Alessia Stornetta, Peter W. Villalta, Hsin-Che Huang, Daniel A. Bachovchin, Silvia Balbo, Rachelle Gaudet, Emily P. Balskus

**Affiliations:** 1 Department of Chemistry and Chemical Biology, Harvard University, Cambridge, MA USA; 2 Department of Molecular and Cellular Biology, Harvard University, Cambridge, MA USA; 3 Masonic Cancer Center, University of Minnesota, Minneapolis, MN USA; 4 Department of Medicinal Chemistry, University of Minnesota, Minneapolis, MN USA; 5 Chemical Biology Program, Memorial Sloan Kettering Cancer Center, New York, NY USA; 6 Division of Environmental Health Sciences, School of Public Health, University of Minnesota, Minneapolis, MN USA; 7 Howard Hughes Medical Institute, Harvard University, Cambridge, MA USA

**Keywords:** Small molecules, Enzymes, Bacteria, X-ray crystallography, Natural products

## Abstract

The human gut bacterial genotoxin colibactin is a possible key driver of colorectal cancer (CRC) development. Understanding colibactin’s biological effects remains difficult owing to the instability of the proposed active species and the complexity of the gut microbiota. Here, we report small molecule boronic acid inhibitors of colibactin biosynthesis. Designed to mimic the biosynthetic precursor precolibactin, these compounds potently inhibit the colibactin-activating peptidase ClbP. Using biochemical assays and crystallography, we show that they engage the ClbP binding pocket, forming a covalent bond with the catalytic serine. These inhibitors reproduce the phenotypes observed in a *clbP* deletion mutant and block the genotoxic effects of colibactin on eukaryotic cells. The availability of ClbP inhibitors will allow precise, temporal control over colibactin production, enabling further study of its contributions to CRC. Finally, application of our inhibitors to related peptidase-encoding pathways highlights the power of chemical tools to probe natural product biosynthesis.

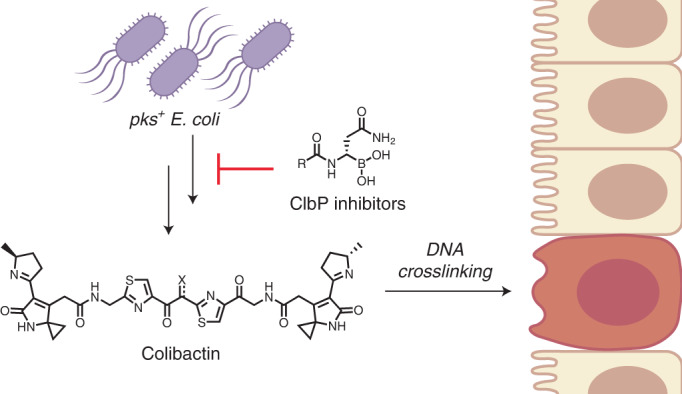

## Abstract (web summary)

A substrate-guided design strategy generated highly potent inhibitors of the biosynthesis of the genotoxin colibactin by human gut bacteria. These inhibitors also enable a generalizable approach for chemically guided natural product discovery.

## Main

The trillions of commensal and pathogenic microorganisms colonizing the human gut, collectively termed the gut microbiota, secrete a diverse milieu of small molecules with profound impacts on human health^1^. In particular, several members of this community are proposed to play roles in the development of CRC, potentially through the production of small-molecule natural products or protein toxins^2,3^. Despite the identification and association of possible microbial culprits with CRC, we still have limited knowledge of the molecular mechanisms underlying the contributions of these organisms to carcinogenesis because of the inherent challenges of studying a complex and dynamic microbial community.

These challenges are exemplified by recent efforts to characterize the genotoxic gut bacterial natural product colibactin. Colibactin is produced by a nonribosomal peptide synthetase–polyketide synthase assembly line encoded by the *pks* genomic island, which is carried by many strains of *Escherichia coli* (*pks*^*+*^ *E. coli*)^4,5^. Although it was initially observed over a decade ago that *pks*^*+*^ *E. coli* elicit a genotoxic phenotype and cause DNA double-strand breaks in cultured epithelial cells, the chemical species responsible for these effects could not be readily identified^4^. Based on extensive metabolomics^6–8^, characterization of biosynthetic enzymes^9^ and total synthesis^10^, potential structures of colibactin have been proposed, although it has not been isolated from a natural source. The proposed structure that is most consistent with colibactin’s DNA alkylating^11^ and crosslinking^12^ activity contains two highly electrophilic cyclopropane warheads linked by a 1,2-diketone; the sensitivity of this linkage to oxidative C–C bond cleavage may explain the difficulty of isolation^9,10^. Formation of the warheads is accomplished in the final step of biosynthesis by the periplasmic peptidase ClbP, which hydrolyzes two units of the *N*-myristoyl-d-asparagine ‘prodrug scaffold’ from a larger pseudodimeric precursor, precolibactin^13,14^. This reaction releases the active species, colibactin, which alkylates two adenine residues on opposite strands of the target cell’s DNA, resulting in highly toxic and mutagenic DNA interstrand crosslinks^10,12^. These crosslinks can break down into monoadducts that are detectable in genomic DNA extracted from cultured cells and epithelial tissue of mice infected with *pks*^*+*^ *E. coli*^11^.

Multiple lines of evidence suggest colibactin-mediated DNA damage plays a role in CRC development. Studies have reported that *pks*^*+*^ *E. coli* are found more frequently in CRC patients relative to healthy controls^15,16^. In addition, colonization with *pks*^*+*^ *E. coli* increased tumor loads in multiple mouse models of colitis-associated CRC^15,17,18^. Multiple studies have reported colibactin-dependent mutational signatures in colonic epithelial cell-derived organoid models, cell lines and in sequenced human cancer genomes. These signatures show a strand bias consistent with interstrand crosslink formation and suggest that colibactin could directly cause CRC driver mutations^19,20^. In cell line models, even short-term exposure to *pks*^*+*^ *E. coli* in culture can cause mutations that lead to changes in growth factor dependence and differentiation in murine colon cells^21^. However, colibactin-dependent mutational signatures were also found in biopsies of morphologically normal colon crypts and were shown to accumulate primarily before the individual reached 10 years of age^22^. Thus, although colibactin is strongly correlated with CRC, the timing and duration of the colibactin insult is likely an important variable in determining CRC risk and is still poorly understood.

Although studies have illuminated a great deal about colibactin’s carcinogenic potential, they also underscore that this natural product and its biosynthetic enzymes are part of a complex network of interactions that cannot be untangled with current tools. Deletion of any of the biosynthetically essential *pks* island genes abolishes production of colibactin, although different deletion mutants in the same strain background result in different phenotypes in vivo, suggesting other roles for these enzymes^23,24^. Genes in the *pks* island have also been linked to a number of seemingly disparate functions including siderophore biosynthesis and microcin production^25,26^. Because the genotoxic effects of colibactin are cell-contact and inflammation dependent, it is challenging to distinguish its effects from other changes in the gut microbiota and host metabolism that occur during periods of inflammation^15,27^. *pks*^*+*^ *E. coli* may also reshape the gut community directly by selectively inhibiting the growth of certain pathogens^28^, forming interspecies biofilms^18^ and inducing prophages in other bacteria^29^.

Investigating these questions requires examining colibactin’s effects in the context of a complex gut community, which is difficult to accomplish with genetic tools. Whole-gene deletions may alter other pathways in which the gene product plays a structural role, and there is evidence that ClbP performs additional, noncatalytic functions in other biosynthetic pathways^26^. Genetic knockouts cannot offer temporal control, making it impossible to probe the importance of timing in colibactin exposure. Controlling exposure using direct addition of colibactin is currently impossible due to its instability and unresolved identity. A tool compound that specifically inhibits colibactin production would shed light on these questions by enabling studies of colibactin in complex *pks*^*+*^ communities with greater precision than is currently possible.

To address this need, we designed and characterized a series of boronic acid-based inhibitors of colibactin biosynthesis. These inhibitors directly engage the colibactin-activating peptidase ClbP and show a high degree of selectivity over unrelated human and bacterial hydrolases. We show that inhibition of ClbP using these compounds abrogates colibactin production in *pks*^*+*^ *E. coli* and *pks*^*+*^ communities. We confirm that these inhibitors completely block the genotoxic effects of colibactin on mammalian cells in culture. Finally, we show that these compounds can also inhibit closely related peptidases found in biosynthetic pathways from soil bacteria, illustrating that this strategy can be generalized to discover biosynthetic intermediates and study the biological roles of other recalcitrant natural products from genetically intractable organisms. By establishing precise control over natural product production, these inhibitors present a unique opportunity to study the effects of colibactin and other natural products in complex microbial communities and explore whether blocking colibactin production could be a viable therapeutic strategy.

## Results

### Synthesis and in vitro testing of putative ClbP inhibitors

To design a specific inhibitor of colibactin biosynthesis, we targeted the colibactin-activating enzyme ClbP. This membrane-embedded periplasmic serine peptidase is essential for the genotoxicity of *pks*^*+*^ *E. coli*. Genetic deletion of ClbP results in an accumulation of biosynthetic intermediates and shunt metabolites from the nonribosomal peptide synthetase–polyketide synthase assembly line, termed ‘candidate precolibactins,’ many of which have been structurally characterized^30,31^. This enzyme is an attractive target for inhibition as it belongs to the same enzyme family as β-lactamases like AmpC, which have been successfully targeted with small molecule inhibitors^13,32^. However, ClbP is not inhibited by several known β-lactamase inhibitors or broad-spectrum serine hydrolase inhibitors in vitro^33^. Although one study identified a pair of boronic acid-based ClbP inhibitors using in silico screening^34^, we found that these compounds have minimal impact on ClbP’s catalytic activity in vitro or in bacterial culture^33^. Thus, there are currently no potent and/or selective inhibitors of this enzyme available.

**Fig. 1:**
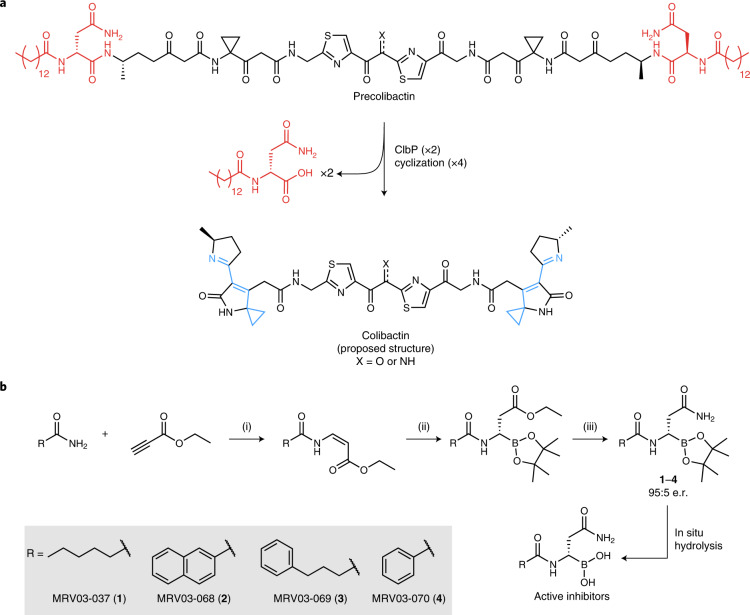
Activity of ClbP guides rational design of colibactin biosynthesis inhibitors. **a**, ClbP activates colibactin by removing an *N*-myristoyl-d-Asn prodrug scaffold (red). Hydrolysis of two amide bonds by ClbP leads to the formation of two electrophilic warheads (cyan) capable of DNA alkylation. Inhibitor design was guided by the two key recognition features of the prodrug scaffold: a d-Asn side chain, which is essential, and a lipid group, which can be modified. **b**, Synthesis of ClbP inhibitors: (i) Pd(OAc)_2_, NaOAc, trifluoroacetic acid, PhMe, 80 °C, 12 h; (ii) CuCl (0.1 equivalent), (*R*)-SEGPHOS (0.11 equivalent), bis(pinacolato)diboron (1.1 equivalents), KO*t*-Bu (1 equivalent), MeOH (4 equivalents), tetrahydrofuran, 3 h; (iii) NaCN (0.2 equivalent), NH_3_, MeOH, 1 h. e.r., enantiomeric ratio.

ClbP contains a catalytic triad typical of the S12 family of serine peptidases formed by the essential residues S95, K98 and Y186 (ref. ^13^). We aimed to target S95 by exploiting ClbP’s essential and unusual acyl-d-Asn substrate-recognition motif (Fig. 1a). Boron-based electrophiles have frequently been employed in inhibitors of serine and threonine peptidases^35^, but posed a synthetic challenge here because of the proximity of the potentially nucleophilic asparagine side chain. We ultimately accessed a small panel of pinacol boronate esters: MRV03-037 (**1**), MRV03-068 (**2**), MRV03-069 (**3**) and MRV03-070 (**4**), which are precursors of the corresponding boronic acids, using an enantioselective copper-catalyzed hydroboration reaction of an ester intermediate followed by ammonolysis (Fig. 1b)^36^.

**Fig. 2:**
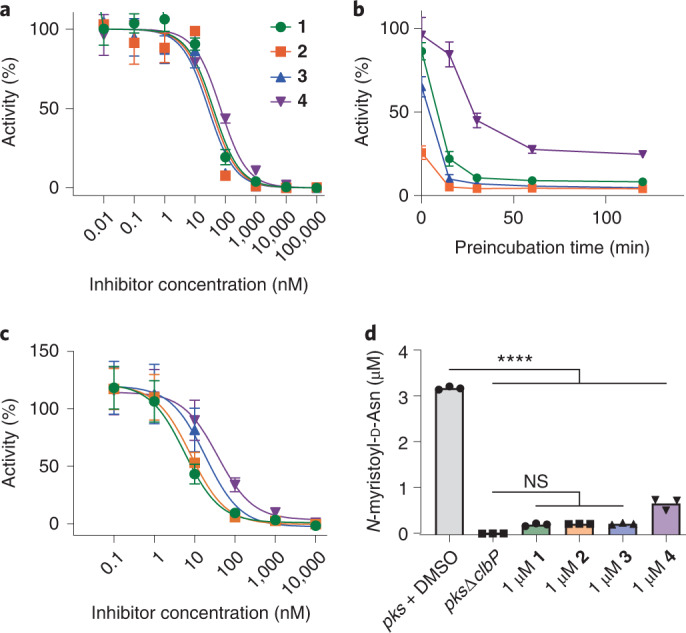
Compounds **1**–**4** inhibit ClbP activity. **a**, ClbP activity in vitro upon treatment with **1**–**4** using a fluorescent substrate analog. **b**, ClbP activity in vitro upon treatment with **1**–**4** at 100 nM and varying incubation time before initiating the assay. **c**, Activity of *E. coli* overexpressing ClbP toward a fluorescent substrate analog after treatment with **1**–**4**. For panels **a**–**c**, symbols show mean value of *n* = 4 biological replicates for each condition, error bars are 1 s.d. **d**, LC–MS measurement of *N*-myristoyl-d-asparagine released from BW*pks* after treatment with vehicle or **1**–**4**. *n* = 3 biological replicates. ****P < 0.0001; not significant (NS), *P* > 0.05, one-way ANOVA and Dunnett’s multiple comparison test. See Methods for calculation of percent activity in fluorescence assays. Source data

**Extended Data Fig. 1:**
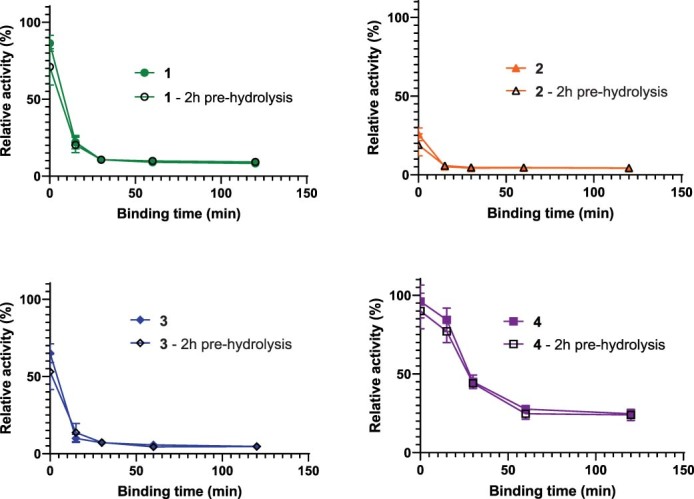
Pinacol ester hydrolysis is not the rate-limiting step of ClbP inhibition. Compounds **1**-**4** show typical ‘slow binding kinetics’ profiles, with potency increasing with longer incubation times and reaching a maximum at approximately 1 hour. When the inhibitors were presoaked in aqueous buffer, no change to this slow binding behavior is observed, indicating that this behavior is not a result of slow hydrolysis of the pinacol boronic ester, but of interaction with ClbP. Each condition was tested in n = 3 biological replicates. Symbols represent the mean of 3 replicates, error bars represent 1 standard deviation (s.d.). Source data

This panel of compounds was initially evaluated using an in vitro fluorogenic ClbP activity assay^33^. With 25 nM purified ClbP, all compounds had median inhibitory concentration (IC_50_) values between 20 and 80 nM after 1 h preincubation, indicating similar potency (Fig. 2a and Supplementary Table 1). Because these compounds were designed to inhibit ClbP by forming a covalent bond with the critical active site serine residue, we examined the kinetics of inhibition using this assay. Highly potent boronic acid-based inhibitors often exhibit slow-binding kinetics, where the free inhibitor is in equilibrium with the noncovalently bound inhibitor–enzyme complex and the subsequent covalent bond-forming step is rate-limiting^37^. As expected for this type of binding, preincubating 100 nM **1**–**4** with ClbP for varying amounts of time confirmed that longer preincubation leads to increased potency (Fig. 2b). Although **1**–**3** reached maximum potency within 30 min, **4** showed a substantial lag, suggesting that the initial noncovalent complex of **4** with ClbP is weaker. Previous work showed that ClbP substrates with smaller acyl groups exhibit higher *K*_M_ values, so the relatively smaller phenyl substituent of **4** may explain this difference^33^. Further control experiments ruled out boronate ester hydrolysis as being rate-limiting (Extended Data Fig. 1).

Using *E. coli* overexpressing ClbP and the same fluorescent reporter yielded IC_50_ values in the range of 4–40 nM for compounds **1**–**4**, indicating that the *E. coli* outer membrane does not impede inhibitor efficacy (Fig. 2c and Supplementary Table 1). To confirm that these inhibitors prevent cleavage of precolibactin by ClbP, we treated a laboratory strain carrying the *pks* island on a bacterial artificial chromosome (*E. coli* BW25113 BAC*pks*, ‘BW*pks*’) with **1**–**4** or a DMSO control and quantified the amount of *N*-myristoyl-d-Asn released by liquid chromatography–mass spectrometry (LC–MS; Fig. 2d). All compounds decreased the quantity of prodrug released, but compound **4** showed slightly lower potency than the others in this format.

### Establishing the mechanism of ClbP inhibition

**Fig. 3:**
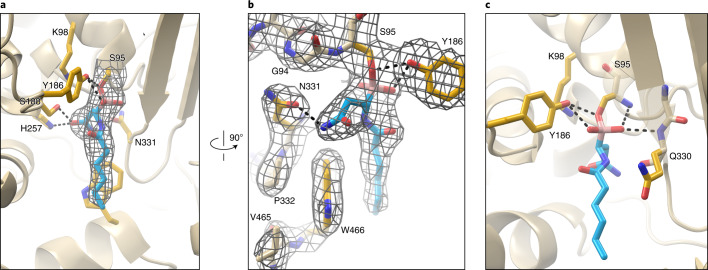
Compound 1 binds the catalytic serine of ClbP directly. **a**, A 2.7-Å resolution structure of ClbP crystallized in the presence of **1** shows the compound (cyan sticks) bound in the expected pocket of the active site near the catalytic triad. Continuous electron density in the polder difference map contoured at 7σ (gray mesh) indicates the inhibitor is covalently bound to S95. **b**, A 90° rotation relative to **a** details the 2F_o_–F_c_ density map contoured at 1σ for the inhibitor and proximal residues. N331 mediates recognition of the d-Asn side chain. **c**, The boronate ester, a structural mimic of the tetrahedral intermediates in colibactin hydrolysis, is stabilized by hydrogen bonds from Q330 and Y186.

To directly examine the interaction between an inhibitor and ClbP, we obtained a crystal structure of full-length ClbP bound to **1** because it bears the same type of aliphatic linear hydrocarbon tail as the native prodrug scaffold (PDB 7MDC, Supplementary Table 2). In this structure, the d-Asn side chain of the inhibitor projects into a tight-fitting pocket and hydrogen bonds with the side chains of residues S188, H257 and N331 (Fig. 3a). N331 also hydrogen bonds to E92, setting its orientation and helping to enforce ClbP’s selectivity for d-Asn- over d-Asp-containing substrates. Other enzyme–substrate interactions and features of interest in the complete structure of ClbP are detailed in a companion paper^38^.

The inhibitor-bound structure of ClbP also reveals continuous electron density from the S95 side chain to the ligand (Fig. 3a), indicating essentially complete conversion to a covalent protein–inhibitor complex. This boronate complex is a structural analog of the tetrahedral intermediates formed during amide bond hydrolysis and participates in several stabilizing interactions that are likely relevant to the activation of precolibactin. The backbone amides of Q330 and S95 hydrogen bond to one of the boronate oxygen atoms and are thus well positioned to stabilize the negative charge that accumulates upon formation of the covalent bond with S95. In addition, Y186, which is part of the catalytic triad that defines this enzyme family, is positioned to stabilize this complex by donating a hydrogen bond to either the other boronate oxygen atom or the oxygen nucleophile on the serine side chain (Fig. 3c). Thus, the potency of these ClbP inhibitors arises both from their ability to mimic the hydrogen-bonding interactions of intermediates in the hydrolysis of precolibactin and the formation of a covalent bond with the catalytic serine residue.

### Selectivity of ClbP inhibitors

**Fig. 4:**
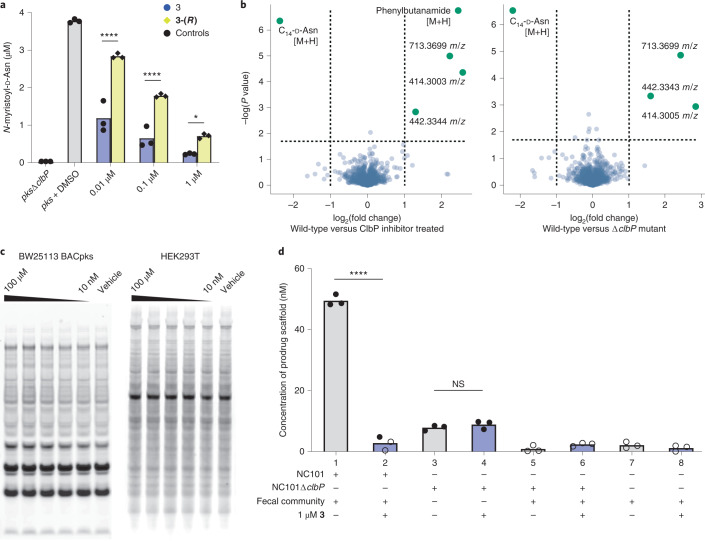
Compound **3** is selective for ClbP inhibition and active in a community setting. **a**, LC–MS measurement of *N*-myristoyl-d-Asn released from BW*pks* after treatment with **3** or **5**. *n* = 3 biological replicates, individual replicates shown. **b**, Volcano plot representation of metabolites detected by LC–MS that are altered in BW*pks* treated with 1 µM of compound **3** versus untreated (left) and BWΔ*P* versus BW*pks* (right). Previously characterized colibactin precursor metabolites are labeled with their *m*/*z*. *n* = 5 biological replicates for all conditions. **c**, Gel-based ABPP using a FP-biotin probe to examine the reactivity of serine hydrolases in *E. coli* and HEK293T cell lysates does not identify any major targets of compound **3**. ClbP is not detected in this assay because of a lack of interaction between it and FP. **d**, LC–MS measurement of the prodrug scaffold in extracts of *E. coli* NC101 and *E. coli* NC101Δ*clbP* cultured with and without compound **3** and with or without a community of organisms resuspended from fecal pellets of C57BL/6J mice. The levels of prodrug scaffold observed in conditions 3 and 4 are expected as a side product from the upstream enzymes in colibactin biosynthesis. *n* = 3 biological replicates for all conditions, individual replicates shown. Empty circles are below the limit of quantitation for this protocol (4 nM). ****P < 0.0001; *P < 0.05; NS, *P* > 0.05; one-way ANOVA and Bonferroni’s multiple comparison test. Source data

**Extended Data Fig. 2:**
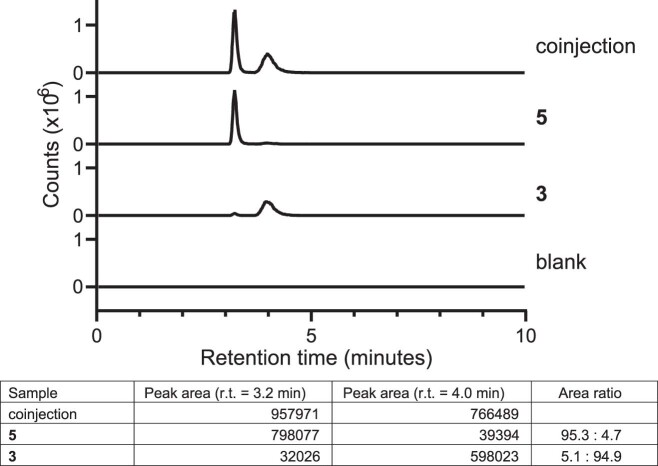
Chiral LC-MS for 3 and 5. Curves shown are extracted ion chromatograms (EICs) for *m*/*z* 361.2308 (+/− 5 ppm) corresponding to the [M + H]^+^ ion of **3** and **5**. The area under the curves (AUCs) for the peaks corresponding to each enantiomer indicate that each compound is a 95:5 molar ratio of major vs. minor enantiomer. Source data

Having established that this group of compounds can bind and inhibit ClbP with high potency, we next focused on establishing the selectivity of these interactions. Because our structural studies suggest that ClbP can only accept inhibitors of this class with the *S* stereochemical configuration, we selected a high-potency inhibitor, **3**, and synthesized the opposite enantiomer, MRV03-095 (**5**). We hypothesized that **5** would be a much less potent inhibitor given that *N*-acyl-l-Asn-containing substrates are not accepted by ClbP^14,33^. Indeed, **5** is 40-fold less potent than **3** at inhibiting the release of the prodrug from BW*pks* (Fig. 4a). The weak inhibitory effect of **5** likely results from the presence of a small amount of **3** as an expected minor product of our synthetic route. Both compounds were prepared in an enantiomeric ratio of 95:5 (determined by chiral LC–MS, Extended Data Fig. 2) for the desired versus undesired enantiomer.

We next used metabolomics to investigate whether treatment with **3** could elicit the same metabolic changes in *E. coli* as a *clbP* genetic knockout. An ideal chemical probe should block colibactin biosynthesis while minimally disrupting other metabolic functions, leading to an accumulation of the same shunt metabolites that have previously been observed in *pks*^*+*^ Δ*clbP* mutant strains. We compared the metabolites produced by BW*pks* with the metabolites produced by an isogenic Δ*clbP* mutant (*E. coli* BW25113 BAC*pks*Δ*clbP*, ‘BWΔ*P*’) using LC–MS (Fig. 4b)^39^. We conducted the same experiment comparing BW*pks* treated with 1 µM **3** versus the DMSO-treated culture (Fig. 4b). The inhibitor-treated wild-type strain displayed similar changes in metabolite abundance as the Δ*clbP* mutant. The primary features identified in both cases were decreased levels of the prodrug scaffold (observed 343.2609 [M+H], theoretical 343.2591 [M+H] mass-to-charge ratio (*m*/*z*)) relative to the DMSO-treated wild-type and an accumulation of known shunt metabolites from colibactin biosynthesis (precolibactins *m*/*z* 414.3004, *m*/*z* 442.3344 and *m*/*z* 713.3699)^6–8^. One other significant feature, an increase of a metabolite at *m*/*z* 164.1055, was observed only in the case of inhibitor treatment and is consistent with the mass of phenylbutanamide (theoretical 164.1070 [M+H] *m*/*z*), a likely degradation product of **3**. Based on these observations, **3** appears to be sufficiently potent and specific to serve as a tool for precise control of ClbP activity in living organisms.

**Extended Data Fig. 3:**
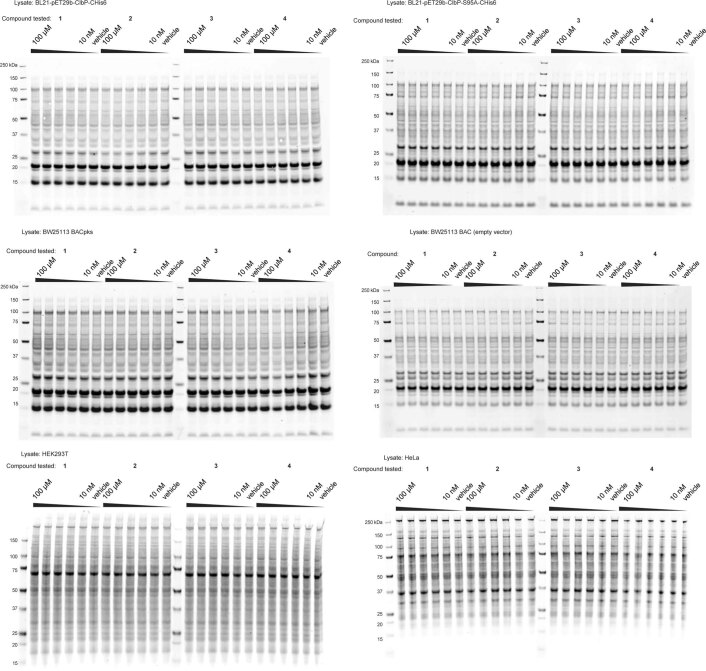
Complete gel images for serine hydrolase ABPP. For all lysates tested, no other clear targets among serine hydrolases could be identified as indicated by the uniform labeling by the fluorophosphonate probe of cellular hydrolases in presence or absence of the tested compounds. Experiments were conducted twice with similar results.

To explore other possible targets of inhibitors **1**–**4**, we used an activity-based protein profiling (ABPP) approach to broadly assay their activity against serine hydrolases in bacterial and mammalian proteomes. In this gel-based assay, the binding of a small molecule to a target protein is detected as a decrease in that protein’s ability to bind a nonspecific fluorophosphonate (FP) probe compound which irreversibly inhibits a wide variety of serine hydrolases^40^. We observed no visible changes in protein labeling by FP at inhibitor concentrations of up to 100 µM in either *E. coli* or HEK293T cell lysates (Fig. 4c and Extended Data Fig. 3). One limitation of this assay is that ClbP is not labeled by the FP probe, so we cannot observe it as a reference for binding. However, this type of assay has been widely used to study the specificity of small molecule–protein binding interactions and provides strong evidence that most serine hydrolases do not bind inhibitors **1**–**4**^41^.

**Extended Data Fig. 4:**
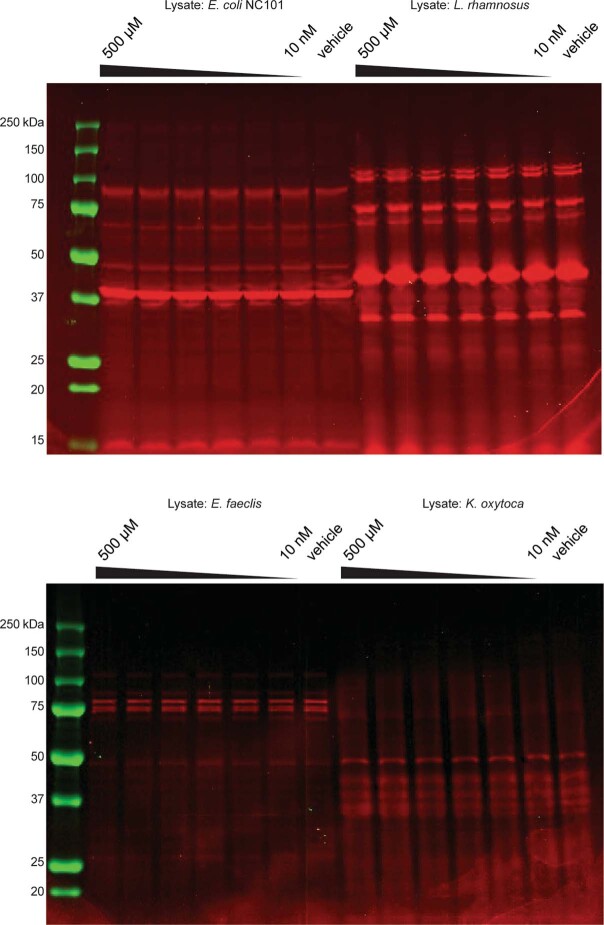
Complete gel images for BOCILLIN-FL ABPP. Gel-based ABPP with the BOCILLIN-FL probe labels known PBPs in *E. coli* NC101 (top left), *L. rhamnosus* (top right), *E. faecalis* (bottom left), and *K. oxytoca* (bottom right) lysates, and inhibitor **3** at concentrations of 10 nM to 500 µM does not inhibit labeling of these proteins. Experiments were conducted twice with similar results.

To further assess the specificity of our inhibitors, we used another ABPP assay in which we replaced the broad-spectrum FP probe with a probe that specifically targets penicillin-binding proteins (PBPs), BOCILLIN-FL. We chose this probe because of ClbP’s homology to AmpC β-lactamases^13^. Because β-lactamases and PBPs both recognize and bind β-lactams, we reasoned that these enzymes are more likely to be secondary targets of our inhibitors. Although this labeling strategy detected several PBPs in various bacterial lysates, inhibitor **3** did not reduce the labeling of any targets (Extended Data Fig. 4). This suggests that the acyl-d-asparagine recognition motif is highly specific to ClbP and its closest homologs.

### Compound 3 inhibits colibactin-associated genotoxicity

**Extended Data Fig. 5:**
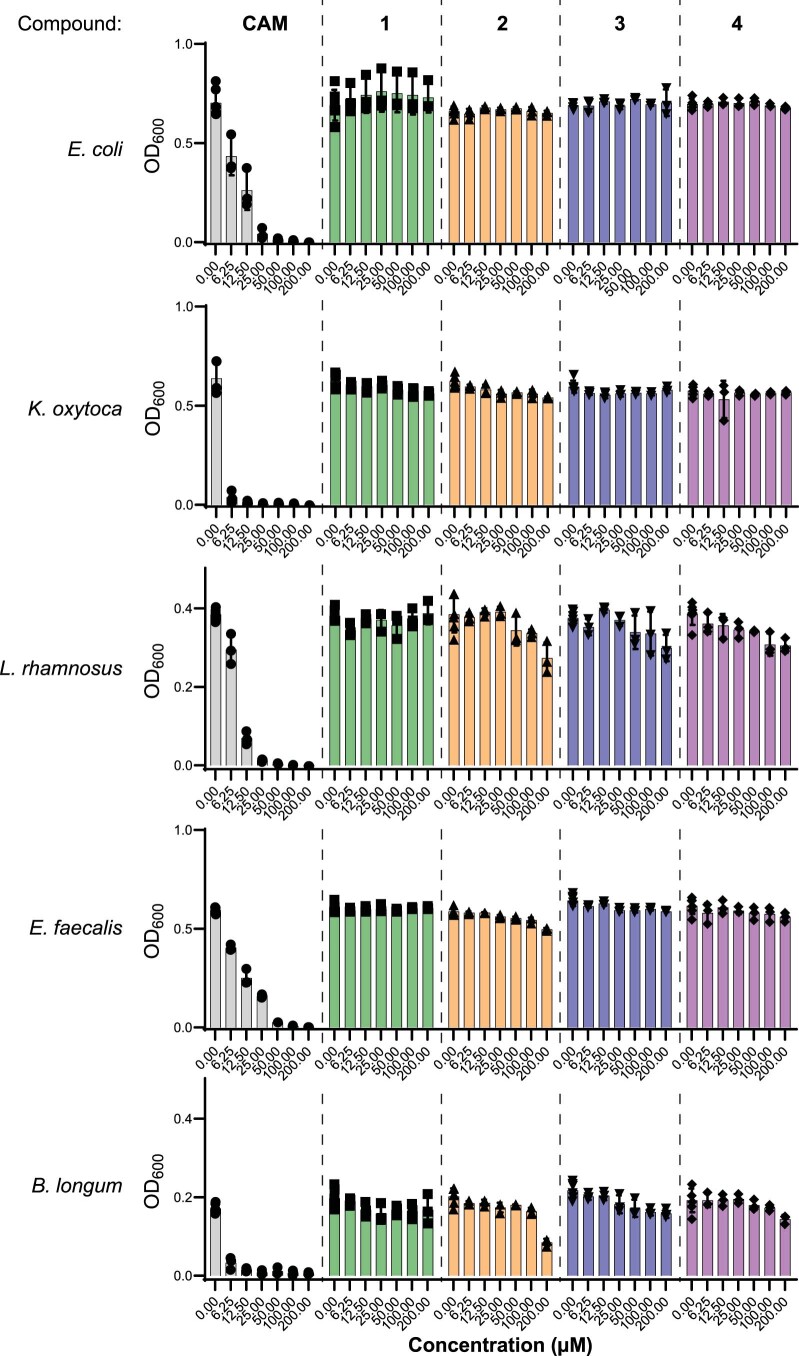
Complete results for MIC determination assays with bacteria. Raw OD_600_ measurements after 15 hours of anaerobic growth for strains tested in MIC assays with compounds **1-4** and chloramphenicol (CAM). Values shown are after subtraction of a media-only blank. Each combination of strain, compound, and concentration was tested in n = 3 biological replicates, with individual replicates shown. Bars show the mean for each condition, error bars are 1 s.d. A summary of these results is given in Supplementary Table 3. Source data

Because colibactin is produced in the context of the gut microbiota, we need to understand how **3** can affect other members of this community and whether it remains an effective inhibitor under these conditions. We simulated a complex *pks*^*+*^ community by inoculating anaerobic liquid cultures with fecal pellets from C57BL/6J mice from the Jackson Laboratory, which do not contain colibactin-producing organisms, and adding *E. coli* NC101 to this community^27^. NC101 is a colibactin-producer isolated from a mouse gut for which an isogenic *E. coli* NC101Δ*clbP* mutant is available as a control^42^. We treated both the simulated *pks*^*+*^ and *pks*Δ*clbP* gut communities with **3** or DMSO and monitored production of the colibactin prodrug scaffold in these cultures by LC–MS. Treatment with **3** fully suppressed production of the prodrug scaffold in the *pks*^*+*^ community to the same level as observed in the *pks*Δ*clbP* community (Fig. 4d). To evaluate the effects of **3** on other members of the gut microbiota, we determined the minimum inhibitory concentrations (MICs) of **1**–**4** against various bacterial strains from common gut phyla, including *E. coli* (Supplementary Table 3). In all cases, MICs were above the upper limit tested in this assay (200 µM), although some species showed partial growth inhibition at 200 µM (Extended Data Fig. 5). The maximum concentration tested here is more than 100-fold greater than the IC_50_ value for inhibition of prodrug release, suggesting that **1**–**4** can be used at concentrations that effectively inhibit ClbP without damaging other members of the gut microbiota.

**Fig. 5:**
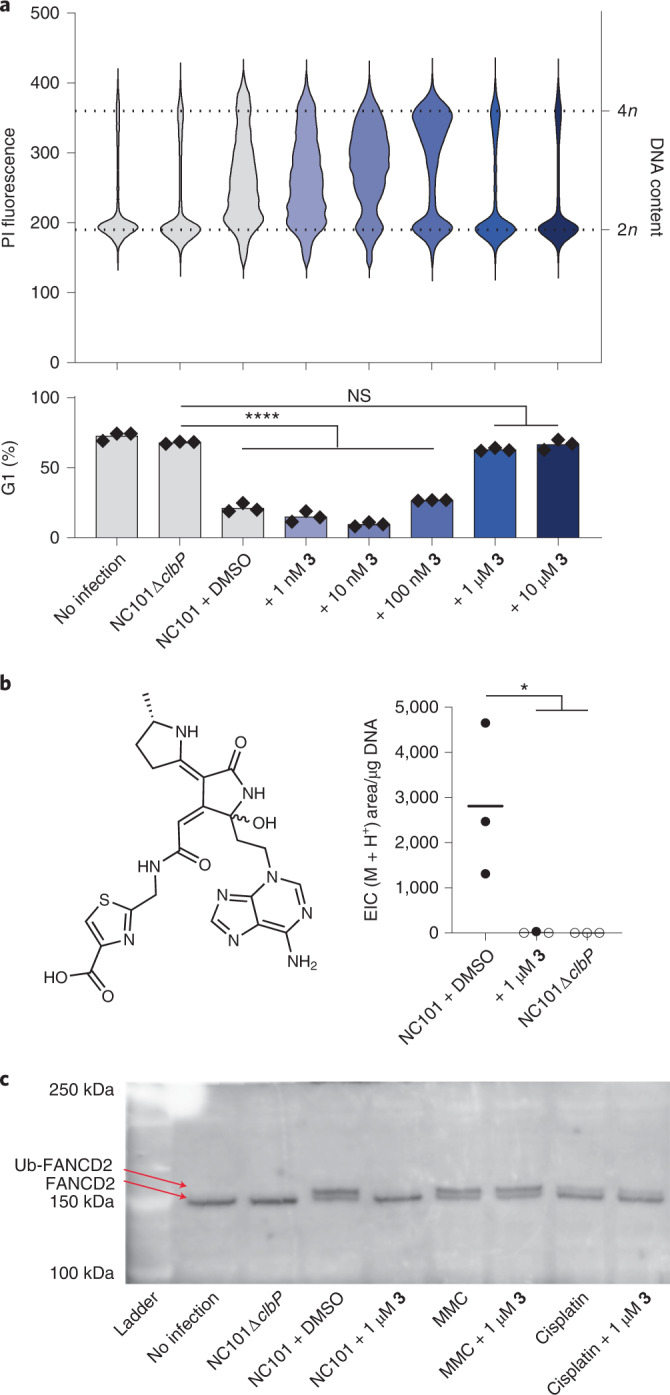
Compound **3** prevents colibactin-induced genotoxicity in human cells. **a**, Flow cytometry analysis of HeLa cells infected with NC101 and treated with **3**. Cells were fixed in ethanol before staining with propidium iodide (PI) for DNA content. The increase in the population fraction with >2*n* DNA content indicates cell-cycle arrest. Top: raw histograms for PI fluorescence intensity in the population for one representative sample for each condition. Bottom: percentage of the population in G1 phase based on fitting histograms to the Watson cell-cycle analysis model^50^. Black symbols are individual replicates, bars show average value. All conditions were run in three biological replicates. Shading indicates the concentration of inhibitor. Gating strategy for flow cytometry is shown in Extended Data Fig. 7. **b**, Structure of two diastereomeric DNA adducts known to be derived from colibactin^11^. LC–MS detection of these adducts (M+H^+^ = *m*/*z* 540.1772) in hydrolyzed genomic DNA extracted from HeLa cells infected with NC101, three biological replicates are shown. Empty circles indicate sample was below the detection limit. For **a** and **b** ****P < 0.0001; *P < 0.05; NS, *P* > 0.05; one-way ANOVA and Dunnett’s multiple comparison test. **c**, Western blot for FANCD2 in HeLa cell extracts. All conditions were run in three biological replicates with one representative sample shown. Source data

**Extended Data Fig. 6:**
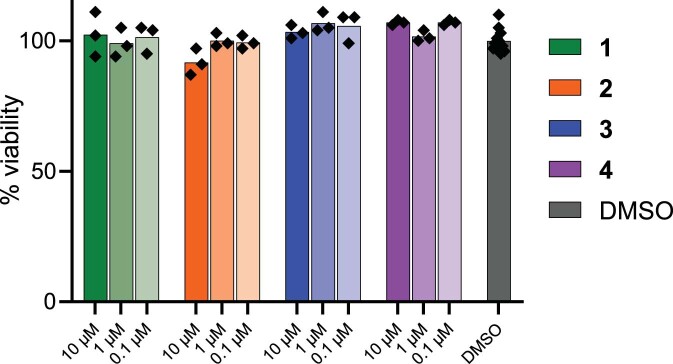
Complete results for MIC determination assays with human cell lines. **1**-**4** do not show cytotoxic activity toward mammalian cell lines up to 10 µM after 20 hours of exposure. No statistically significant difference was observed between any of the conditions tested (n = 3 biological replicates for each) and the DMSO control (n = 12) using an ordinary one-way ANOVA and Dunnett’s multiple comparison test (*p* > 0.05 in all comparisons to DMSO control). Source data

In addition to blocking the metabolic indicators of colibactin biosynthesis, we also assessed whether **3** could inhibit the genotoxic effects of colibactin on human cells. Cells exposed to colibactin exhibit cell-cycle arrest which can be quantified by DNA staining and flow cytometry^4^. We exposed HeLa cells to NC101 (ref. ^15﻿]^) and added **3** at varying concentrations in the infection medium. Treatment with **3** partially inhibits this effect at 100 nM (Fig. 5a), with complete inhibition at 1 µM. We also confirmed that **3** is not cytotoxic to human cell lines at all concentrations tested (≤10 µM, Extended Data Fig. 6).

To directly assess the impacts of **3** on colibactin’s DNA alkylating activity, we infected HeLa cells with NC101 with or without **3** added and isolated their genomic DNA. This DNA was hydrolyzed and analyzed by LC–MS to detect two diastereomeric colibactin-derived DNA adducts (Fig. 5b)^11^. Treatment with 1 µM **3** suppressed adduct formation similarly to genetic deletion of *clbP*. Finally, we assessed the impact of **3** on the response to colibactin-mediated DNA damage in HeLa cells. In response to stalled replication forks caused by DNA crosslinks, the protein FANCD2 is monoubiquitinated (FANCD2-Ub)^43^, and cell lines missing FANCD2 show increased sensitivity to colibactin^12^. Using a western blot, we detected an increase in abundance of FANCD2-Ub in HeLa cells in response to exposure to NC101, as well as in response to the DNA crosslinking agents mitomycin C (MMC) and cisplatin (Fig. 5c). Treatment of cells with 1 µM **3** prevented FANCD2 ubiquitination in cells exposed to colibactin but not MMC or cisplatin, indicating that **3** is specific to the colibactin biosynthetic pathway and does not inhibit the DNA damage response. Thus, compound **3** is not only an inhibitor of ClbP, but a potent and specific inhibitor of colibactin’s genotoxicity.

### Compound 3 is a tool for natural product characterization

**Fig. 6:**
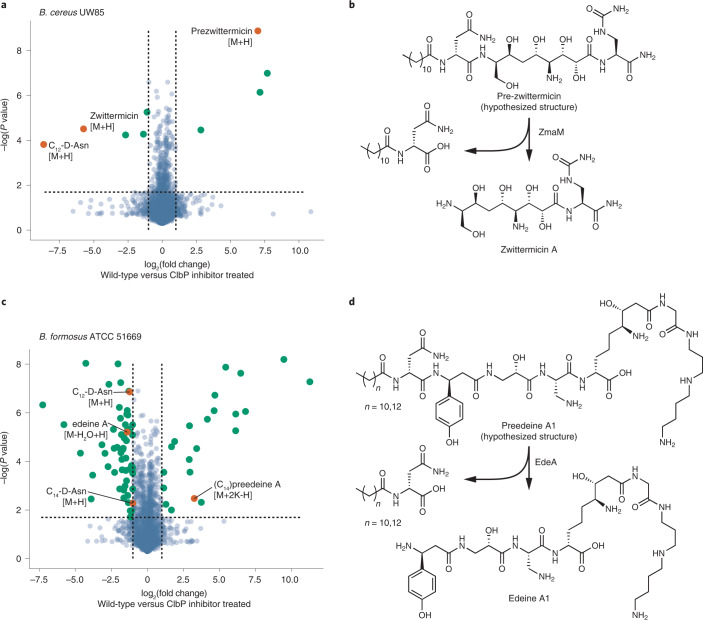
Compound 3 can be used to manipulate other natural product biosynthesis pathways. **a**,**c**, Comparative metabolomic analysis of culture supernatants of (**a**) *B. cereus* UW85 and (**c**) *B. formosus* ATCC 51669 treated with inhibitor **3** versus untreated. Inhibitor treatment leads to a decrease in production of zwittermicin or edeines, respectively, with an accumulation of precursor metabolites in both cases. Selected mass features associated with each natural product are highlighted in orange. Significance calculated using one-sided Student’s *t*-test. **b**,**d**, Schemes for the reported and proposed prodrug activation steps in (**b**) zwittermicin and (**d**) edeine A1 biosynthesis, respectively, which are consistent with the observed metabolomic shifts. A complete list of the mass features identified in the experiment with *B. cereus* UW85 showing significant changes can be found in Supplementary Table 4, and those from *B. formosus* ATCC 51669 can be found in Supplementary Table 5.

Finally, we explored the generality of this approach for controlling biosynthetic pathways by inhibiting other prodrug-activating peptidases (Fig. 6). Because compounds **1**–**4** exploit ClbP’s unusual *N*-acyl-d-asparagine recognition motif, we expected them to also inhibit ClbP’s closest relatives which hydrolyze similar substrates. Although no ClbP homologs have been annotated outside of the *pks* gene cluster in organisms from the human gut microbiota, they are widely distributed in biosynthetic gene clusters from environmental bacteria^38^. As genetic deletion of *clbP* causes an accumulation of upstream biosynthetic intermediates and shunt products, characterization of which was key to the structural elucidation of colibactin^6–8^, we reasoned that **3** could be used in a similar fashion to identify other natural products biosynthesized using an *N*-acyl-d-asparagine-mediated prodrug activation step. This could accelerate the discovery and structural characterization of such targets without the need to develop new genetic tools in each host organism which encodes a ClbP homolog.

**Extended Data Fig. 8:**
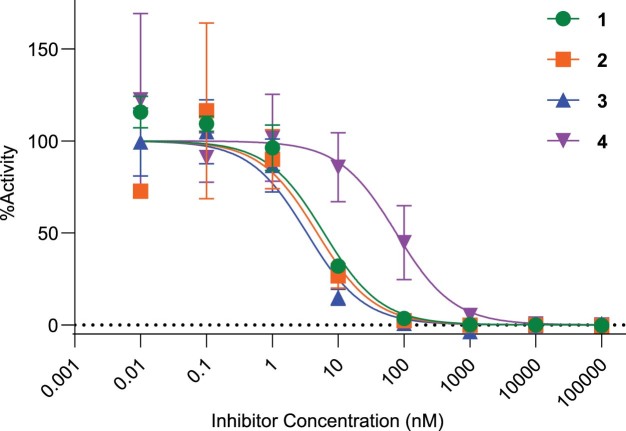
Compounds 1–4 inhibit ZmaM cleavage of a fluorogenic probe *in vitro*. Normalized activity in an in vitro fluorescence assay of purified ZmaM treated with **1-4** or vehicle. n = 3 biological replicates for each condition, symbols represent the mean, error bars are 1 s.d. Source data

**Extended Data Fig. 9:**
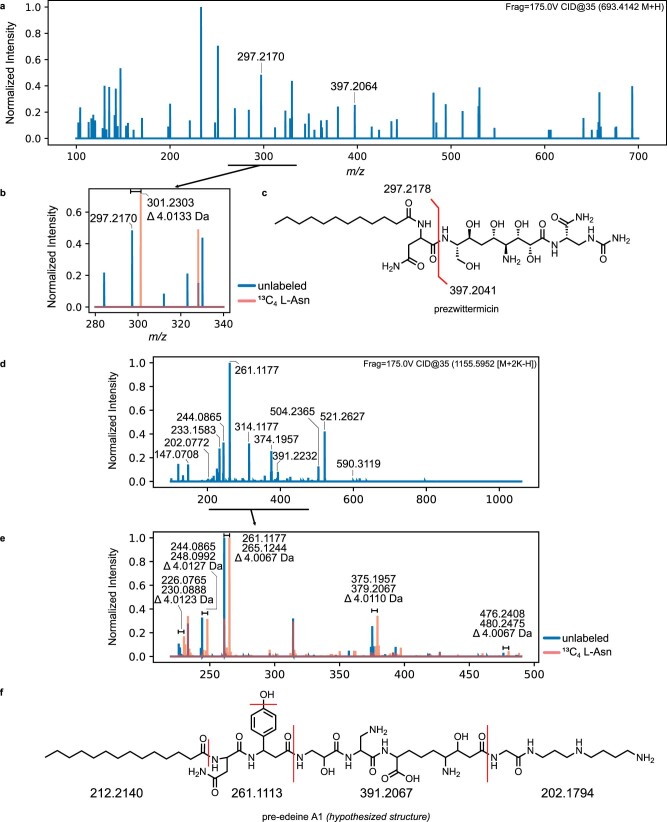
MS/MS fragmentation and isotopic labeling of proposed prezwittermicin and preedeine A. **(a)** Daughter ion spectrum of prezwittermicin metabolite 693.4142 *m*/*z*. **(b)** Zoom in on 280–340 *m*/*z* region of **(a)**, with the daughter ion spectrum of ^13^C_4_-labeled prezwittermicin (697.4275 *m*/*z*) overlaid in orange. The proposed *N*-lauroyl-d-asparagine fragment shows the expected +4 *m*/*z* shift. **(c)** Fragmentation pattern of the key fragments highlighted in **(a)** and **(b)**. **(d)** Daughter ion spectrum of preedeine metabolite 1155.5952 *m*/*z*. **(e)** Zoom in on 200–490 *m*/*z* region of **(d)**, with the daughter ion spectrum of ^13^C_4_-labeled preedeine (1159.6011 *m*/*z*) overlaid in orange. Several daughter ions show the +4 *m*/*z* shift, which can be attributed to the fragmentation events summarized in **(f)**. These data do not distinguish between the 1 and the 2 isomer. The 1 isomer is shown in part (f) for illustrative purposes only.

We first tested this approach by targeting ZmaM, a ClbP homolog that is proposed to activate the antibiotic zwittermicin^44^. Though zwittermicin biosynthesis was the first pathway proposed to use this type of prodrug mechanism, the precursor prezwittermicin has not yet been identified. As expected, **1–4** inhibit purified ZmaM with similar potency to ClbP in vitro (Extended Data Fig. 8). We compared the metabolite profiles of untreated cultures of the zwittermicin producer *Bacillus cereus* UW85 and cultures exposed to **3** using LC–MS. Treatment with **3** caused an accumulation of a species whose mass is consistent with the proposed structure of prezwittermicin (observed 693.4136, theoretical 693.4141 [M+H] *m*/*z*, Fig. 6a). In addition, masses consistent with both free zwittermicin (observed 397.2046, theoretical 397.2041 [M+H] *m*/*z*) and a free *N*-lauroyl-d-asparagine (observed 315.2279, theoretical 315.2278 [M+H] *m*/*z*) were depleted in the treated cultures (Fig. 6a). MS/MS fragmentation patterns of these ions and their counterparts from cultures fed ^13^C_4_-asparagine support these structural assignments (Fig. 6c and Extended Data Fig. 9). The successful detection of prezwittermicin highlights the capabilities of an inhibitor-guided discovery approach.

We then applied this strategy to study edeines, a family of natural products whose biosynthetic gene cluster encodes a ClbP homolog, but whose production has never been reported to involve a prodrug resistance mechanism^38,45^. We treated the edeine producer *Brevibacillus formosus* ATCC 51669 with **3** and observed a depletion of several masses corresponding to edeines, as well as masses consistent with both *N*-lauroyl- and *N*-myristoyl-d-asparagine (Fig. 6b). There are currently four known members of the edeine family (A, B, D and F), each of which has two naturally occurring structural isomers (1 or 2)^45^. Focusing on edeine A, treatment with **3** resulted in accumulation of a mass consistent with a larger premetabolite incorporating the *N*-myristoyl-d-asparagine motif (observed 1155.5952, theoretical 1155.5940 [M+2K-H] *m*/*z*, Fig. 6b). Employing the same ^13^C_4_-asparagine labeling and MS/MS fragmentation strategy, we observed +4 *m*/*z* shifts in the mass corresponding to this putative premetabolite, as well as consistent with *N*-lauroyl- and *N*-myristoyl-d-asparagine (Fig. 6d and Extended Data Fig. 9). Based on this, we propose that edeine biosynthesis uses a prodrug activation strategy and that the larger mass discovered after treatment with **3** represents a newly discovered ‘preedeine’. The greater number of metabolites that change in abundance after treatment with **3** in *B. formosus* compared with *B. cereus* likely reflects the known structural diversity of edeines. Further adding to this complexity, bioinformatic analyses reported in an accompanying paper identified a gene encoding a second ClbP homolog in *B. formosus* (locus tag BP422_09930 in the CP018145.1 genome), suggesting that it produces another family of uncharacterized natural products using a similar prodrug resistance strategy^38^. It is likely that some of the mass shifts that we observed can be attributed to this cryptic biosynthetic gene cluster. Together, these experiments demonstrate that compound **3** can be used as a tool to discover and study other natural products from diverse environmental microbes.

## Discussion

Leveraging our understanding of colibactin biosynthesis, we developed a panel of compounds that can potently and specifically inhibit the colibactin-activating peptidase ClbP. Past observations that ClbP recognizes a structural motif not commonly found in other metabolites made it well-suited for substrate-guided inhibitor design. Notably, none of the inhibitors tested here show substantial differences in activity in vitro, despite bearing different *N*-acyl structures. This may indicate that the acyl group serves only a weak role in initial substrate recognition, while potency is driven by the reversible covalent bond formed between ClbP’s catalytic serine residue and the boronic acid electrophile. Distinguishing which features are essential for potency and recognition will aid future medicinal chemistry efforts, as well as the design of other chemical tools with additional chemical functionality in the acyl group of the molecule.

In addition to their high potency for ClbP inhibition, these compounds do not appear to interact with any of the other serine hydrolases examined in our assays. A potential risk of using electrophilic inhibitors is that their high potency and slow off-rates will lead to inhibition of many cellular targets. We surveyed a broad range of potential secondary targets, including PBPs in the same β-lactamase family as ClbP, using ABPP assays and did not observe evidence of off-target activity. Metabolomics experiments illustrate that treatment with these compounds does not broadly change metabolism outside of the colibactin biosynthetic pathway. Examining additional aspects of inhibitor selectivity would be aided by the preparation of chemical tools based on **1**–**4** which can form irreversible covalent bonds with ClbP and other potential targets. However, the fact that these compounds show limited toxicity to various organisms, including bacteria and mammalian cells, and minimal metabolic perturbation outside of the colibactin pathway are strong evidence that their off-target effects are low.

One of the most promising aspects of these inhibitors is the opportunity they provide to study colibactin’s effects in the context of a complex *pks*^*+*^ community. We show that **3** can selectively block colibactin biosynthesis in *E. coli* NC101 in the presence of a complex gut community without antibiotic activity toward representatives of major gut bacterial phyla. These experiments also showed that baseline production of the prodrug scaffold is more than tenfold lower when *pks*^+^ *E. coli* are present in low abundance in a community setting versus in monoculture. This dramatic difference is a reminder that studies in which germ-free mice are monocolonized with *pks*^*+*^ bacteria cannot offer a complete picture of colibactin’s impacts in a community context. Small molecule tools like **3** will enable studies to determine whether observations from monocolonization studies can be reproduced in conventional hosts with *pks*^*+*^ communities.

Finally, we confirmed that, in addition to blocking the formation of key metabolites like the prodrug scaffold, **3** can also prevent the genotoxic effects of colibactin on human cells. Treatment with **3** prevents cell-cycle arrest, DNA adduct formation and FANCD2 ubiquitination, biomarkers widely used to monitor colibactin’s genotoxicity^11,12,15^. A major challenge to studying colibactin has been the inability to establish precise control over colibactin exposure, limiting our ability to establish clear, causal connections between this unique toxin and the changes in the host which have been attributed to its activity. Our inhibitors can address this problem and allow us to dissect colibactin’s role in cancer with a new level of detail.

These chemical tools can also be applied beyond the context of colibactin and the gut microbiota. The family of prodrug-activating peptidases related to ClbP is highly selective in substrate scope and widely distributed in biosynthetic gene clusters from environmental bacteria^46^. We showed that our inhibitors can selectively disrupt production of both zwittermicin and the edeines. These experiments allowed us to uncover the biosynthetic intermediate ‘prezwittermicin’, which had only been predicted based on the biosynthetic gene cluster. Moreover, we provide the first experimental observation, to our knowledge, of preedeine metabolites, directly demonstrating how targeted small molecule inhibitors can be used for natural product discovery. We envision the general strategy of combining metabolomics analysis with inhibitors of biosynthetic enzymes may be applied more broadly to enable studies of natural products which are difficult to isolate, synthesize, or study with genetic tools.

Chemical modulation of gut microbial functions is a promising avenue both for therapeutic intervention and enabling basic research into the mechanisms of microbiota-host interactions^47–49^. Applying this strategy to colibactin biosynthesis offers a new way to interrogate the relationships between this enigmatic natural product, the surrounding microbiota, their host and cancer. Using inhibitors like **3**, we can begin to understand how the duration and timing of colibactin exposure influence tumorigenesis. These inhibitors may also help illuminate the connection between colibactin and inflammation, which can be intermittent in the gut, but is essential for colibactin-related CRC in mouse models. In the long term, these molecules may serve as a starting point to evaluate colibactin biosynthesis as a therapeutic target for the prevention or treatment of CRC.

## Methods

For chemical synthesis procedures and compound characterization data, see Supplementary Note. Unless otherwise stated, statistical comparisons and nonlinear curve fitting were conducted in GraphPad Prism 9.

### Bacterial strains

*E. coli* NC101 and *E. coli* NC101Δ*clbP* were provided by the laboratory of C. Jobin (University of Florida Department of Medicine, 2033 Mowry Rd, Office 461, Gainesville, FL 32611-0882, USA). *Klebsiella oxytoca* (ATCC 8724) was obtained from the American Type Culture Collection. *Lactobacillus rhamnosus* strain LMS2-1, *Enterococcus faecalis* strain TX0104 and *Bifidobacterium longum* strain 44B were obtained from the Biodefense and Emerging Infections Research Resources Repository.

### Constructs and protein purification for in vitro assays

ClbP constructs described in this publication were derived from a previously described plasmid containing the *E. coli* CFT073 ClbP sequence (GenBank ID: NP_754344.1) inserted between the NdeI and XhoI restriction sites of pET29b (Addgene plasmid no. 48244)^14^. All in vitro experiments used a construct bearing a C-terminal 10×His tag. This longer polyhistidine-tag was obtained by extension of the previously described 6×His tag through site-directed mutagenesis (forward: CCACCATCACCATCACTGAGATCCGGCTGCTAACAAAGCCCGAAAG, reverse: CTCAGTGATGGTGATGGTGGTGGTGGTGGTGGTGCTCGAGCTC). All site-directed mutations were introduced using the QuikChange mutagenesis protocol (Stratagene) and confirmed by Sanger DNA sequencing of the whole open reading frame. Plasmids were transformed into chemically competent C41(DE3) (Lucigen) cells and proteins were isolated as previously described^33^. The *zmaM* coding sequence from *B. cereus* UW85 (forward: GAAGGAGATATACATATGAAGTTAAACATATGGTTGAAGTTTATCATTTTA, reverse: GTGATGGTGATGATGAGCGGCCGCTAATAATGCCTCCTTTGTTTTATTCATTTTCAC) was inserted between the NdeI and NotI sites of a modified pET21a that introduces a C-terminal 3×Ala linker followed by a 10×His tag. The S89A mutation was introduced by PCR with mismatched primers (forward: GCTAGGTGCTACTTCAAAAGCTTTTACGGCATTAGCTG, reverse: GAAGTAGCACCTAGCTCGAAAAGCGTCTCATTCG) and the coding sequence confirmed with Sanger sequencing.

### ClbP and ZmaM fluorescence activity assays (in vitro)

Assays were performed in a buffer containing 50 mM Tris, 200 mM NaCl, 0.02% w/v *n*-dodecyl-β-d-maltoside (DDM) at pH 8.0 with 25 nM purified enzyme and 25 µM fluorogenic substrate in a total volume of 20 µl^33^. Assays with ZmaM also included 5 mM Mg^2+^ and 1 mM ATP in the assay buffer. Purified enzyme was thawed on ice from stocks stored at –80 °C. Stocks were diluted to 50 nM enzyme in assay buffer in the wells of a black, flat-bottom 384-well plate and the appropriate inhibitor in DMSO or DMSO was added to a final concentration of 1% v/v. For experiments testing different inhibitor concentrations, reactions were allowed to sit for 1 h at room temperature. For experiments testing different preincubation times, the inhibitor in DMSO was added to each reaction X min before initiating the reaction, where X is the time indicated on the *x*-axis for that sample. For experiments to test whether hydrolysis of the boronic ester was rate-limiting, the inhibitor was added to buffer with no enzyme present and allowed to sit at room temperature for the time indicated before adding the enzyme. In all cases, reactions were initiated by the addition of 10 µl of buffer containing the 50 µM fluorogenic substrate to achieve a final concentration of 25 µM and pipetting once with a multichannel pipette to mix. Reaction progress was monitored in a plate reader (Bio-Tek Synergy HTX multimode plate reader) with an excitation filter of 360/40 nm and an emission filter of 440/20 nm. ‘% activity’ was determined based on the measured relative fluorescence units (RFU) of each condition after 1 h using the following formula:

$${{{\mathrm{\% }}}}\,{\mathrm{activity}} = \frac{{{\mathrm{RFU}}_{\mathrm{{sample}}} - {\mathrm{RFU}}_{{\mathrm{ClbP - S95A}}}}}{{{\mathrm{RFU}}_{{\mathrm{vehicle}}} - {\mathrm{RFU}}_{{\mathrm{ClbP - S95A}}}}} \times 100$$

### ClbP fluorescence activity assay (live cells)

One 5-ml starter culture each of *E. coli* BL21 pET-29b-ClbP and pET-29b-ClbP-S95A was inoculated from frozen stocks and grown overnight at 37 °C in lysogeny broth (LB) medium supplemented with 50 μg ml^−1^ kanamycin (LB + kan). Overnight cultures were diluted 1:100 in fresh LB + kan and incubated at 37 °C to an OD_600_ of 0.3, at which point protein expression was induced by the addition of 500 μM isopropyl β-D-1-thiogalactopyranoside (IPTG) and cultures were moved to 15 °C for 4 h. Cultures were then aliquoted in a black 384-well plate and the appropriate concentration of inhibitor was added as a DMSO stock to a final concentration of 1% DMSO in a volume of 30 μl. Reactions were initiated by the addition of 10 μl of LB + kan + IPTG containing the fluorogenic substrate (final concentration 100 μM). Plates were incubated at 25 °C with intermittent shaking while taking regular fluorescence measurements in a Bio-Tek Synergy HTX multimode plate reader with an excitation filter of 360/40 nm and an emission filter of 440/20 nm. ‘% activity’ was determined based on the formula above using RFU measurements after 7 h and 45 min.

### Liquid chromatography–mass spectrometry quantitation of *N-*myristoyl-d-Asn produced by *pks*^*+*^*E. coli*

One 5-ml starter culture each of *E. coli* BW25113 BAC*pks* and *E. coli* BW25113 BAC*pks*Δ*clbP* was inoculated from frozen stocks and grown overnight at 37 °C in LB medium supplemented with 35 μg ml^−1^ chloramphenicol (LB + cam). Overnight cultures were diluted 1:100 in fresh LB + cam + 1% DMSO with the appropriate concentration of inhibitor and grown for 20 h at 37 °C in a deep-well plate in a shaking incubator. A 500-μl aliquot was taken from each sample, flash frozen in liquid nitrogen and lyophilized to dryness. Lyophilized pellets were extracted with 500 μl of LC–MS grade methanol which contained 100 nM d_27_-*N-*myristoyl-d-asparagine as an internal standard, prepared as previously described, and vortexed for 30 s. After centrifugation at 16,500*g* for 10 min in a tabletop microcentrifuge, supernatants were transferred to fresh 1.5-ml tubes and stored at –20 °C overnight. Samples were centrifuged again, and the supernatants analyzed by LC–MS/MS on a Waters Xevo TQ-S UPLC-triple quadrupole mass spectrometer using an Agilent Poroshell 120 EC-C18 column (2.7 mm, 4.6 mm × 50 mm). The conditions were as follows: 0.6 ml min^−1^ flow rate, 5 µl injection, 10% solvent B in solvent A for 1 min, a linear gradient increasing to 90% solvent B in solvent A over 2 min, 90% solvent B in solvent A for 1.5 min, followed by a linear gradient to 2% solvent B in solvent A over 30 s, and re-equilibration at 2% solvent B for 1 min (solvent A, 95:5 water/methanol + 0.03% ammonium hydroxide; solvent B, 80:15:5 isopropanol/methanol/water). The mass spectrometer was run in negative mode MRM with a cone voltage of 2 V, monitoring transitions of *m*/*z* 341 → *m*/*z* 114 (retention time (rt), 3.3 min; collision energy (CE), 20 V) for the prodrug scaffold and *m*/*z* 368 → *m*/*z* 114 (rt, 3.3 min; CE, 22 V) for the deuterated internal standard. Data analysis was conducted using the TargetLynx software platform (Waters) and Microsoft Excel. For all samples, peak areas for the *m*/*z* 341 → *m*/*z* 114 transition were normalized to the *m*/*z* 368 → *m*/*z* 114 transition for the same sample, and then normalized values were compared with a standard curve of unlabeled *N-*myristoyl-d-asparagine containing 100 nM d_27_-*N-*myristoyl-d-asparagine, which was run in triplicate.

### ClbP expression and purification for crystallography

ClbP-6×His was expressed and purified as described^38^. C41(DE3) cells transformed with the construct were grown in terrific broth supplemented with 50 µg ml^−1^ kanamycin until they reached an OD_600_ of 0.6. Cells were induced with 0.5 mM IPTG and grown for 20 h at 15 °C. Cells were harvested through centrifugation at 3,300*g* (Beckman JS4.2 rotor) for 15 min and flash frozen. To isolate the membrane fraction, cells were thawed and resuspended in load buffer (20 mM sodium phosphate pH 8.0, 20 mM imidazole, 500 mM NaCl, 10% glycerol) supplemented with 1 mM PMSF and 1 mM benzamidine. Cells were disrupted by sonication on ice (six cycles of 45 s each in a Branson Sonifier 450 under duty cycle of 65% and output control of 10) and cell debris was cleared from the lysate by centrifugation at 31,000*g* (Beckman JA-20) for 20 min. Membranes were pelleted by ultracentrifugation at 158,000*g* (Beckman type 45Ti) for 70 min, homogenized in load buffer using a glass Potter-Elvehjem grinder, and solubilized by incubation with 1% (w/v) DDM (Anatrace) for 2 h under constant mixing at 4 °C. Detergent-insoluble materials were removed by ultracentrifugation at 95,000*g* (Beckman type 45Ti) for 35 min and the supernatant was incubated with Ni-Sepharose resin (Qiagen) for 2 h under constant mixing. The resin was washed with 12 column volumes (CV) of load buffer containing 0.03% DDM, 10 CV of load buffer containing 0.5% lauryl maltose neopentyl glycol (LMNG; Anatrace), and 12 CV of load buffer containing 0.1% LMNG. ClbP was eluted in two fractions of 6 and 3 CV of load buffer containing 450 mM imidazole and 0.01% LMNG. Both elutions were combined, concentrated and injected onto an S200 10/300 size-exclusion column (GE Healthcare) equilibrated with SEC buffer (10 mM Tris pH 8.1, 150 mM NaCl, 0.003% LMNG). Column fractions enriched with ClbP were pooled, concentrated to 7 mg ml^−1^ in a volume of 450 µl and incubated with a approximately tenfold molar excess of **1** (addition of 11 µl of a 50 mM DMSO stock) on ice for 3 h to allow complete binding. Protein was finally concentrated to 24 mg ml^−1^ and flash frozen.

### Purification of ZmaM for inhibition assays

Protein was expressed and purified as described for ClbP for crystallography, except that all purification buffers contained 0.05% DDM and were supplemented with 5 mM MgCl_2_ and 1 mM ATP. ZmaM was eluted from the Ni affinity resin by stepwise incubation with buffers containing 75, 100, 150, 250, 300 and 450 mM imidazole. The 250 and 300 mM imidazole elutions were pooled and dialyzed overnight against ~200 volumes of 50 mM Tris pH 8.0, 200 mM NaCl, 0.02% DDM, 5 mM MgCl_2_ and 1 mM ATP. Dialyzed ZmaM was concentrated in a 100 kDa centrifugal filter (EMD Millipore) and flash frozen in liquid nitrogen.

### Inhibitor-bound ClbP crystallization

Inhibitor-bound wild-type ClbP was crystallized as described^38^. In short, frozen stocks of wild-type ClbP incubated with **1** were thawed and reconstituted in a monopalmitolein mesophase (1:1 protein to monopalmitolein ratio) using the syringe reconstitution method. The mesophase bolus was dispensed onto custom-made 96-well glass sandwich plates using an NT8 drop setter (Formulatrix) in 75-nl drops and overlaid with 900 nl of precipitant (mixture of 200 nl of 0.1 M imidazole pH 7.8, 10% (v/v) PEG400, 150 mM Li_2_SO_4_ containing 11 mM of **1** and 700 nl of 0.1 M Tris pH 7.2, 25% (v/v) PEG400, 200 mM Li_2_SO_4_). Crystals appeared after 12 h, and were harvested after 7 d using mesh loops (MiTeGen) and plunge freezing in liquid nitrogen.

### Diffraction data collection and processing

Diffraction data were collected at beamline 23ID-B of the Advanced Photon Source at a wavelength of 0.98 Å. Data from a single crystal were indexed using DIALS^51^, scaled in CCP4 AIMLESS^52,53^, and phased by molecular replacement in PHENIX^54^ using the model of full-length ClbP reported in an accompanying paper (PDB: 7MDE) as the search model^38^. Statistical data are listed in Supplementary Table 2.

### Structure refinement and model building

Model building was done in COOT^55^ and refinement was done in Phenix.refine by a series of five macrocycles including reciprocal space refinement, TLS parameters and individual B-factors, and optimizing the X-ray/atomic displacement parameter weights. The final model of inhibitor-bound ClbP contained residues 36–411 and 430–491, with 96.31% of backbone atoms in Ramachandran favored regions, 3.69% in allowed regions and no outliers. Model statistics are listed in Supplementary Table 2.

Structural biology applications used in this project were compiled and configured by SBGrid^56^.

### Chiral liquid chromatography–mass spectrometry

The relative chirality of **3** and **5** was determined using an Agilent Technologies 1200 series LC equipped with a Phenomenex Lux 5 mm Amylose-1 column (100 × 4.6 mm). Compounds were eluted in an isocratic mobile phase of 5% water with 0.1% formic acid/95% acetonitrile with 0.1% formic acid (flow rate 0.5 ml min^−1^; injection volume 2 ml). Compounds were detected using an Agilent 6530 Q-TOF Mass Spectrometer fitted with a dual-spray ESI source. The capillary voltage was set to 3.5 kV, the fragmentor voltage to 175 V, the skimmer voltage to 65 V and the Oct1 RF to 750 V. The drying gas temperature was maintained at 275 °C with a flow rate of 8 l min^−1^ and a nebulizer pressure of 241 kPa. A standard calibrant mix was introduced continuously during all experiments via the dual-spray ESI source in positive mode. Masses corresponding to the [M+H]^+^ ions (± 5 ppm) of **3** and **5** were extracted using the Qualitative Analysis software platform and integrated to determine the area under the curve for each analyte. Retention times were confirmed by coinjection of the two compounds mixed 1:1, and a dilution series of both compounds was run to ensure that the area under the curve for these compounds was linear with respect to concentration of compound.

### Metabolomics

One 5-ml starter culture each of *E. coli* BW25113 BAC*pks* and *E. coli* BW25113 BAC*pks*Δ*clbP* was inoculated from frozen stocks and grown overnight at 37 °C in LB medium supplemented with 35 μg ml^−1^ chloramphenicol (LB + cam). Overnight cultures were diluted 1:1,000 in fresh LB + cam + 1% DMSO with or without 1 μM inhibitor **3** in five replicates for each condition and grown for 20 h at 37 °C. A 500-μl aliquot was taken from each replicate, flash frozen in liquid nitrogen and lyophilized to dryness. Lyophilized pellets were extracted with 500 μl of LC–MS grade methanol and prepared in the same manner as described above for metabolomics. Samples were analyzed by LC–MS on an Agilent Technologies 1200 series LC with a Phenomenex Luna C18 column (5 mm, 100 Å, 250 × 4.6 mm) coupled to an Agilent 6530 quadrupole-time of flight mass spectrometer. The following chromatography conditions were used: 99% solvent A in solvent B for 1.5 min, linear gradient to 0% solvent A in solvent B over 43.5 min, 0% solvent A for 8 min, linear gradient back to 99% solvent A over 1 min, equilibration in 99% solvent A for 9 min at a flow rate of 0.4 ml min^−1^ and a 10-μl injection volume. Solvent A is water with 0.1% formic acid, solvent B is acetonitrile with 0.1% formic acid. Mass spectrometry was conducted in ESI+ mode, with a source gas flow of 8 l min^−1^ at 275 °C, capillary voltage of 3,500 V, fragmentor at 175 V, skimmer at 65 V and Oct1 RF at 750 V.

Studies of both *B. cereus* and *B. formosus* were conducted in a similar manner with the following modifications. Cultures were inoculated from single colonies in 0.5× TSB for 72 h at 30 °C. Additional culturing conditions included 1 μM of inhibitor **3** and/or the addition of 1 mM ^13^C_4_-l-asparagine (Cambridge Isotopes). After incubation, cultures were filtered through a 0.2-μm membrane and separated on a Phenomenex Kinetex C_18_ 100 × 3 mm column coupled to the same Agilent 6530 quadrupole-time of flight mass spectrometer. The chromatographic method begins at a composition of 98% solvent A for 2 min, a linear gradient to 40% solvent A over 23 min, a further linear gradient to 5% solvent A over 5 min then holding at 5% solvent A for 2.5 min, at a flow rate of 0.4 l min^−1^.

Data analysis was performed using MzMine 2.53 (ref. ^39^). Features were detected using the ADAP chromatogram builder algorithm^57^. Significance between treatment groups was calculated by a Student’s *t*-test with a minimum of *n* = 3 or *n* = 5 when available.

### General ABPP of serine hydrolases

Bacterial or mammalian cell lysates were normalized to 1 mg ml^−1^ protein concentrations in PBS using the DC protein assay (Bio-Rad). Aliquots (50 µl) of lysates were incubated with compounds at the indicated concentrations (1 µl of a 50× stock in DMSO) for 30 min at room temperature and subsequently labeled with 5 µM of fluorophosphonate-PEG(4)-biotin probe (1 µl of a 50× stock in DMSO, synthesized as previously reported) for 1 h at room temperature. The reactions were mixed with 50 µl of 2× SDS protein loading buffer, boiled for 10 min at 95 °C, and separated by SDS–PAGE. The labeled proteins were detected by IR dye-conjugated streptavidin (Li-Cor) and visualized using the GellOdyssey Imaging System (Li-Cor)^58^.

### ABPP of PBPs

Cultures of the indicated bacterial species were grown overnight at 37 °C in either 8 ml of LB (*E. coli*), MRS (*L. rhamnosus*) or Wilkins–Chalgren (*E. faecalis* and *K. oxytoca*) liquid media. Cells were pelleted by centrifugation at 12,000*g* at 4 °C for 10 min. Supernatants were removed and masses of wet cell pellets were recorded. Each pellet was then resuspended in 6 µl of buffer per mg of cell pellet (lysis buffer: 50 mM Tris, 100 mM NaCl, pH 7.2, 1× Bug Buster lysis reagent, 1 µl ml^−1^ benzonase nuclease, 1 mg ml^−1^ lysozyme). Resuspensions were incubated at room temperature for 30 min with gentle shaking, then centrifuged briefly in a bench top centrifuge to remove large debris (lysates were not clarified). Lysates were treated with the indicated concentrations of inhibitor in 20-µl reactions at room temperature for 1 h before 25 µM BOCILLIN-FL was added and all reactions were incubated at 4 °C overnight. Then 20 µl of 2× SDS–PAGE loading buffer containing DTT was added, followed by heat denaturing at 70 °C for 10 min. Samples were run on Novex WedgeWell 10%–20% Tris–glycine PAGE gels at 100 V for 2–2.5 h before imaging on an Azure Sapphire Biomolecular Imager.

### Bacterial minimum inhibitory concentration assay

Minimum inhibitory concentrations (MICs) of compounds against different bacterial species were determined using a modified version of a broth microdilution protocol, which has been previously reported^59^. Briefly, cultures of each species indicated were inoculated from frozen stocks in deoxygenated Wilkins–Chalgren anaerobic medium inside an anerobic chamber with an 92.5% N_2_/5% CO_2_/2.5% H_2_ atmosphere. Cultures were grown for 24–48 h in a 37 °C incubator at which point an OD_600_ measurement was taken for each culture and which was accordingly diluted in fresh Wilkins–Chalgren anaerobic medium to an equivalent of OD_600_ of 0.01. Diluted cultures were then distributed into the wells of a clear, flat-bottom 384-well plate and the compound of interest was added to a final DMSO concentration of 2% (or DMSO only for positive controls). Then 10 ml of mineral oil (Millipore Sigma) was deposited on top of the cultures and the plates were covered with a clear adhesive plate seal. Plates were incubated at 37 °C for 16 h, at which point the OD_600_ of all wells was recorded on a SpectraMax M2 plate reader (Molecular Devices). All conditions were tested in triplicate, and the MIC was determined as the minimum concentration of inhibitor at which the culture showed a statistically significant decrease (*p* < 0.05, one-way analysis of variance (ANOVA) followed by Dunnett’s multiple comparison test) in OD_600_ compared with the DMSO-only control of the same species at the same time point.

### ClbP inhibition in a microbial community

Mouse fecal pellets were aseptically collected from cages and immediately stored at –80 °C until use. One 5-ml starter culture each of *E. coli* NC101 and *E. coli* NC101Δ*clbP* was started from frozen stocks in deoxygenated Brain–Heart Infusion medium inside an anaerobic chamber under a 95% N_2_/5% H_2_ atmosphere and incubated at 37 °C overnight. Mouse fecal pellets were thawed in the anaerobic chamber and resuspended in Brain–Heart Infusion medium with 10 ml of medium per 100 mg of pellet mass. The fecal slurry was then centrifuged at 1,000*g* for 5 min to separate solids and the resulting supernatant was used as the media for all ‘+community’ conditions. Cultures were incubated in 500-μl volumes in triplicate in a deep-well plate under anaerobic conditions, with a 1:100 inoculum of the appropriate *E. coli* overnight for the ‘+*E. coli*’ conditions. All samples contained a final concentration of 1% DMSO, with or without 1 μM inhibitor **3**. After 20 h at 37 °C, samples were processed, and the concentration of *N-*myristoyl-d-asparagine was quantified via LC–MS/MS as described above.

### General tissue culture methods

HeLa cells (ATCC CCL-2) were maintained at 37 °C in a humidified 5% CO_2_ incubator using Gibco DMEM (Thermo Fisher Scientific) supplemented with 10% FBS (Thermo Fisher Scientific) and penicillin/streptomycin/amphotericin B cocktail (Thermo Fisher Scientific). Cell stocks were passaged every 3 d at a 1:4 split ratio.

### HeLa cell survival assay

HeLa cells were maintained as described in the General Tissue Culture Methods section. After trypsinization and counting, cells were distributed into the wells of a 96-well plate with media containing the indicated concentration of the synthetic compound and 1% DMSO (or DMSO alone) at 5,000 cells per well. After 20 h, cell viability was determined using the CellTiter-Glo 3D Cell Viability Assay kit (Promega) following the manufacturer’s instructions and measuring luminescence on a Bio-Tek Synergy HTX multimode plate reader.

### Infection with *pks*^*+*^*E. coli*

Assays for cell-cycle arrest and FANCD2 ubiquitination were performed by seeding 24-well plates with 125,000 HeLa cells per well and incubating those plates under standard conditions for 24 h. At the same time, cultures of NC101 and NC101Δ*clbP* were started from frozen stocks in LB medium and grown at 37 °C with shaking overnight. The next day, bacterial overnights were diluted 1:50 into fresh tissue culture media that did not contain antibiotics (DMEM + FBS). Cultures were monitored until an OD_600_ of 0.3–0.5 was reached. At that point, samples of the cultures were removed and DMSO stock solutions of the inhibitor of interest were added to each sample to a final concentration of 1% DMSO and the indicated concentration of inhibitor.

Media in the 24-well plate was aspirated, HeLa cells were washed with sterile DPBS (Thermo Fisher Scientific), and fresh DMEM + FBS + 1% DMSO with the indicated concentration of inhibitor was added. A volume of *E. coli* equivalent to 2.5 × 10^7^ bacteria based on OD in DMEM + FBS + 1% DMSO with the indicated concentration of inhibitor was then added directly to wells (multiplicity of infection 1:100). Infections were carried out for 4 h at 37 °C in a humidified 5% CO_2_ incubator. Media was then aspirated and cells were washed twice with DPBS to remove bacteria and fresh DMEM + FBS + PSF supplemented with 50 µg ml^−1^ gentamicin was added.

### Cell-cycle analysis

Between 20 and 24 h after infection, HeLa cells were trypsinized (0.25% Trypsin-EDTA, Gibco), washed with DPBS, and fixed in cold 70% ethanol and stored at 4 °C until flow cytometry analysis (24–48 h). Cells were centrifuged at 800*g* for 10 min and 70% ethanol was aspirated. Cells were resuspended in DPBS for 15 min, then centrifuged and supernatant aspirated again. Cells were then resuspended in DPBS with 0.2 mg ml^−1^ RNAse A (Invitrogen) and 0.02 mg ml^−1^ propidium iodide (PI; Millipore Sigma). After 30 min, cells were analyzed on a BD LSR II Analyzer at the Harvard University Bauer Core Flow Cytometry Facility. For each replicate, 10,000 events were collected and results were gated for single cells and plotted using the FloJo software package.

### DNA adduct detection

Infections for DNA adduct detection were carried out using the protocol described above in six-well plates in which all volumes and cell numbers were increased by a factor of four accordingly. After trypsinization, cell pellets were frozen at –80 °C until analysis. DNA was isolated from cells as previously reported^11^. DNA samples (100 µl, 31–48 μg) in silanized glass vials were incubated at 80 °C for 1 h. After incubation, samples were allowed to cool to room temperature, and volumes were increased to 200 µl by adding LC–MS grade water. Samples were filtered using a Centrifree Ultrafiltration Device (relative molecular mass of 30,000 (*M*_r_ 30K), Millipore Sigma) at 2,000*g* for 15 min. Samples were dried under vacuum and stored at –20 °C. Mass spectrometric data were acquired with the following conditions. The dried samples were reconstituted in 10 µl of H_2_O and 4 µl of the resulting solution was injected onto an UltiMate 3000 RSLCnano UPLC (Thermo Fisher Scientific) system equipped with a 5 μl injection loop. Separation was performed with a capillary column (75 μm internal diameter, 20 cm length, 10 μm orifice) created by hand packing a commercially available fused-silica emitter (New Objective) with 5 μm Luna C18 bonded separation media (Phenomenex). The flow rate was 1,000 nl min^−1^ for 5.5 min at 0% CH_3_CN, then decreased to 300 nl min^−1^ followed by a linear gradient of 0.05% formic acid aqueous solution of 3.57% per min over 7 min. The column was washed at 95% CH_3_CN for 2 min and re-equilibrated at 0% CH_3_CN with a flow rate of 1,000 nl min^−1^ over 2 min. The injection valve was switched at 6 min to remove the sample loop from the flow path during the gradient. Mass spectrometric data were acquired with an Orbitrap Lumos mass spectrometer (Thermo Fisher Scientific). Positive mode electrospray ionization was used under nanospray conditions (300 nl min^−1^) using a Thermo Scientific Nanoflex ion source with a source voltage of 2.2 kV. The instrument was operated with a capillary temperature of 300 °C and an S-Lens RF level setting of 60%. Targeted MS/MS spectra of the *m*/*z* 540.1772 analyte were acquired with a quadrupole isolation window of *m*/*z* 1.5 centered on *m*/*z* 540.2 with an HCD fragmentation setting of 25%, resolution setting of 120,000, normalized AGC target of 2,000%, and maximum injection times of 1,000 ms. All spectra were acquired with the EASY-IC lock mass (*m*/*z* 202.0777) enabled.

### Western blot for FANCD2

Infections were carried out as described above. For conditions that involved other small molecules, no bacteria were introduced during the infection and cells were treated with either 0.5 µg ml^−1^ MMC or 7.5 µg ml^−1^ cisplatin under the same conditions for 4 h. At 20 h after infection, cells were lysed directly in the culture wells (lysis buffer: 50 mM Tris, pH 8.0, 150 mM NaCl, 1% Triton X-100, 0.5% sodium deoxycholate, 0.05% SDS, 1 mM MgSO_4_, SIGMAFAST protease inhibitor mix, 100 U ml^−1^ benzonase (Millipore Sigma)). Lysates were then spun down at 16,000*g* to remove particulates and the supernatant was mixed 1:1 with 2× Laemmli sample buffer (Bio-Rad) containing 50 mg ml^−1^ DTT. Samples were then heated at 70 °C for 10 min and run on an SDS–PAGE gel (3%–8% Novex Tris-Acetate Gel, Invitrogen) at 150 V for 1.5 h at 4 °C. Bands were then transferred to PVDF membranes (100 V, 1 h at 4 °C) and blocked with 10% skim milk powder in TBST buffer at 4 °C overnight. Blots were probed sequentially with either mouse anti-actin (1.5 µg ml^−1^) or mouse anti-FANCD2 (0.1 µg ml^−1^) primary antibodies (Thermo Fisher Scientific, catalog nos. MA511869 and MA123347), followed by a peroxidase-conjugated goat anti-mouse secondary antibody (Jackson ImmunoResearch, catalog no. 115-035-003; 0.08 µg ml^−1^). Each probing step was conducted for 90 min at room temperature n 2.5% skim milk powder in TBST. Before imaging, blots were washed 3 times for 15 min each in TBST. Blots were imaged using the SuperSignal West Pico PLUS Chemiluminescent Substrate (Thermo Fisher Scientific) kit following the manufacturer’s instructions and imaged using an Azure Biosystems c300 imager.

### Reporting summary

Further information on research design is available in the Nature Research Reporting Summary linked to this article.

## Online content

Any methods, additional references, Nature Research reporting summaries, source data, extended data, supplementary information, acknowledgements, peer review information; details of author contributions and competing interests; and statements of data and code availability are available at 10.1038/s41589-022-01147-8.

## Supplementary information

Supplementary Information Supplementary Tables 1–5, including IC_50_ values for inhibitors (1), crystallographic statistics (2), MIC values for inhibitors against bacteria (3), and significant features identified by metabolomics for *B. cereus* and *B. formosus* (4 and 5); Note containing synthetic procedures and characterization data for compounds 1–5.

Reporting Summary

## Figures and Tables

**Extended Data Fig. 7:**
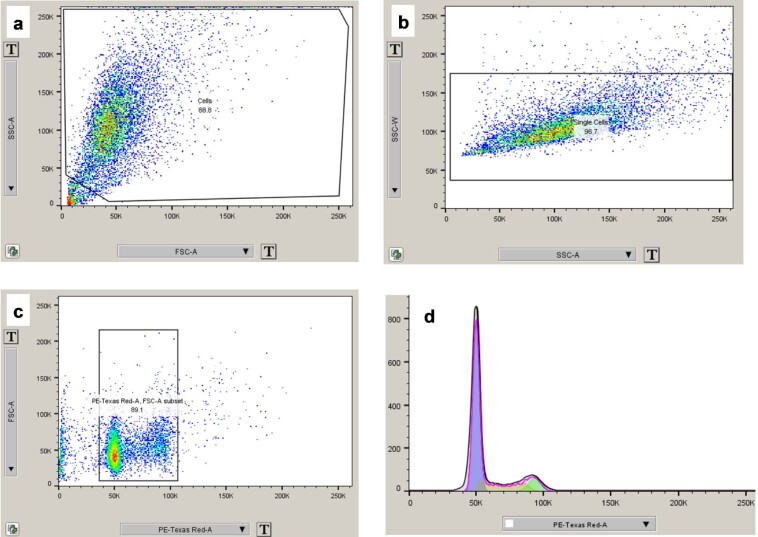
Gating strategy for flow cytometry experiments. Images captured directly from FloJo 10.7.1. **(a)** After collecting at least 10,000 events for each sample, each sample was gated on a plot of FSC-A vs SSC-A to separate debris from cells (retaining 70–90% of events). **(b)** This cell population was then gated on SSC-W vs SSC-A to separate single cells (91–98%). **(c)** The single cell population was then gated on FSC-A vs propidium iodide fluorescence to remove unstained cells and other outliers (89–97%). **(d)** This final population was then plotted as a histogram and fit to the Watson Model. In some cases, noise in the data made automatic unconstrained fitting impossible, and the fitting process was aided by constraining the G1 peak center to the left (less fluorescent) half of the population and setting the condition that the G2 peak CV = G1 peak CV. In all cases, the same gate values were applied to every sample at each stage of gating.

## Data Availability

Atomic coordinates and structure factors for the reported crystal structure in this work have been deposited to the Protein Data Bank under accession number 7MDC. Corresponding X-ray diffraction images have been deposited to the SBGrid Data Bank under accession number 832 (10.15785/SBGRID/832). Flow cytometry data have been deposited in the Flow Repository and are publicly accessible at http://flowrepository.org/id/FR-FCM-Z5M5. Mass spectrometry data are available via the Harvard Dataverse at 10.7910/DVN/0UIFRP. Source data are provided with this paper.

## Source data

Source Data Fig. 2 Plate reader (fluoresce) and LC–MS peak area data for panels a–d (each panel its own tab).
Source Data Fig. 4 LC–MS peak area data for panels a and d. Additional Source Data for panels in Fig. 4 available online. See Data Availability Statement.
Source Data Fig. 5 Uncropped chemiluminescence and visible light images of Western blots for FANCD2. Additional Source Data for panels in Fig. 5 available online. See Data Availability Statement.
Source Data Extended Data Fig. 1 Plate reader (fluoresce) measurements.
Source Data Extended Data Fig. 2 Raw counts for chiral LC–MS analysis of compounds **3** and **5**.
Source Data Extended Data Fig. 5 Raw OD_600_ measurements of bacterial cultures used in MIC assays.
Source Data Extended Data Fig. 6 Raw luminescence measurements used to determine HeLa cell viability.
Source Data Extended Data Fig. 8 Raw fluorescence measurements for ZmaM cleavage of a fluorogenic substrate upon treatment with **1**–**4**.
