# Supplementary material for: A small molecule inhibitor prevents gut bacterial genotoxin production
Source: Nat Chem Biol. 2022 Oct 17;19(2):159–67. doi: 10.1038/s41589-022-01147-8 (PMC9889270; doi:10.1038/s41589-022-01147-8)
Supplement: Supplementary file 1 — Supplementary Tables 1–5, including IC50 values for inhibitors (1), crystallographic statistics (2), MIC values for inhibitors against bacteria (3), and significant features identified by metabolomics for B. cereus and B. formosus (4 and 5); Note containing synthetic procedures and characterization data for compounds 1–5. [file 41589_2022_1147_MOESM1_ESM.pdf]

nature chemical biology

Article

https://doi.org/10.1038/s41589-022-01147-8

A small molecule inhibitor prevents gut bacterial genotoxin production

In the format provided by the authors and unedited

Supplementary Information

A small molecule inhibitor prevents gut bacterial genotoxin production

Matthew R. Volpe[1], José A. Velilla[2], Martin Daniel-Ivad[1], Jenny J. Yao[1], Alessia Stornetta[3], Peter W. Villalta[3,4], Hsin-Che Huang[5], Daniel A. Bachovchin[5], Silvia Balbo[3,6], Rachelle Gaudet[2], Emily P. Balskus[1,7,8]

Affiliations

1 Department of Chemistry and Chemical Biology, Harvard University, 12 Oxford Street, Cambridge, MA 02138, USA

2 Department of Molecular and Cellular Biology, Harvard University, 52 Oxford Street, Cambridge, MA 02138, USA

3 Masonic Cancer Center, University of Minnesota, 2231 Sixth Street Southeast, Minneapolis, MN 55455, USA

4 Department of Medicinal Chemistry, University of Minnesota, 308 Harvard Street Southeast, Minneapolis, MN 55455, USA

5 Chemical Biology Program, Memorial Sloan Kettering Cancer Center, New York, NY, USA

6 Division of Environmental Health Sciences, School of Public Health, University of Minnesota, Minneapolis, MN 55455, USA

7 Howard Hughes Medical Institute, Harvard University, 12 Oxford Street, Cambridge, MA 02138, USA

8 To whom correspondence should be addressed (balskus@chemistry.harvard.edu)

Supplementary Table 1– IC50 values of **1**-**4** measured both *in vitro* and in *E. coli* using a fluorogenic assay, with 95% confidence interval values given in parentheses. All assays were conducted with *n* = 4 biological replicates and normalized dose-response data were fit to a non-linear three-parameter model.

Compound	IC_{50} measured *in vitro*<br><br>(95% confidence interval)	IC_{50} measured in *E. coli* BL21 overexpressing ClbP<br>(95% confidence interval)
**1**	40 nM (30 – 54 nM)	5.6 nM (4.0 – 7.9 nM)
**2**	34 nM (22 – 54 nM)	7.2 nM (5.2 – 10.0 nM)
**3**	28 nM (19 – 40 nM)	18.3 nM (12 – 28 nM)
**4**	69 nM (52 – 92 nM)	27 nM (18 – 38 nM)

Supplementary Table 2 - Data collection and refinement statistics (molecular replacement) for PDB: 7MDC

	WT ClbP bound to **1** (PDB: 7MDC)
**Data collection**	
Space group	P 4_2 2_1 2
Cell dimensions	
a , *b* , *c* (Å)	96.72, 96.72, 183.38
α, β, γ (°)	90, 90, 90
Resolution (Å)	45.58 - 2.7 (2.8 - 2.7)
Total reflections	269998 (27526)
Unique reflections	24663 (2389)
I / σI	8.06 (1.54)
R_{sym} or R_{merge}	0.2575 (1.887)
R_{meas}	0.2702 (1.975)
CC1/2	0.996 (0.534)
Completeness (%)	99.88 (99.92)
Redundancy	10.9 (11.5)
Wilson B-factor	48.5
**Refinement**	
Resolution (Å)	45.58 - 2.7 (2.8 - 2.7)
No. reflections	24644 (2387)
No. reflections in R_{free}	1231 (118)
R_{work} / R_{free}	0.1926 / 0.2359
No. atoms	3635
Protein	3315
Ligand/ion	108
Water	212
B -factors	
Protein	57.74
Ligand/ion	75.99
Water	50.75
R.m.s. deviations	
Bond lengths (Å)	0.007
Bond angles (°)	0.94
Ramachandran plot	
Favored (%)	96.31
Allowed (%)	3.69
Disallowed (%)	0
Rotamer outliers (%)	2.63
Clashscore	5.71

Supplementary Table 3 – Minimum inhibitory concentrations (MICs) of compounds against other human-associated bacteria. Chloramphenicol (CAM) was included as a control for antibiotic activity. MICs were determined using a broth dilution assay (data shown in Extended Data Figure 5) and measuring OD600 values after 15 hours of growth compared to a DMSO control. Values reported here are the lowest concentration of compound at which a statistically significant ($p < 0.05$, one-way ANOVA and Dunnett's multiple comparison test, n = 3 biological replicates) difference in turbidity was observed. "≤6.25" indicates that significant growth inhibition was observed even at the lowest concentration tested; ">200" indicates that no significant growth inhibition was observed at any concentration tested.

	MICs by compound (μM)				
Species	CAM	**1**	**2**	**3**	**4**
Escherichia coli NC101	25	>200	>200	>200	>200
Klebsiella oxytoca	6.25	>200	>200	>200	>200
Lactobacillus rhamnosus	25	>200	>200	>200	>200
Enterococcus faecalis	50	>200	>200	>200	>200
Bifidobacterium longum	12.5	>200	>200	>200	>200

Supplementary Table 4 – Complete list of metabolite ions from LC-MS metabolomics experiments with *B. cereus* UW85 which meet selection criteria (p -value < 0.02 calculated using one-sided Students T-test, >2 fold change) for being significantly enriched or depleted when cultures were treated with inhibitor **3**. Dashed lines indicating cutoffs are shown in Figure 6.

	m/z	Retention Time (min)	Fold Change (log2)	Significance (-log(P))	Asn Labeling	Annotation
**Features Enriched without inhibitor**	315.2279	23	-8.673	3.823	Y	*N*-lauroyl-D-Asn [M+H]
	397.2046	1.79	-5.755	4.518		Zwittermicin
	288.1572	2.93	-2.681	4.246		
	202.1793	1.48	-1.376	4.282		
	453.7569	20.04	-1.113	5.266		
**Features Enriched with inhibitor**	709.413	12	2.826	4.468		
	693.4136	17.67	6.996	8.891	Y	Prezwittermicin
	519.3379	20.61	7.151	6.151		
	723.4239	13.58	7.688	7.001		

Supplementary Table 5 – Complete list of metabolite ions from LC-MS metabolomics experiments with *B. formosus* ATCC 51669 which meet selection criteria (*p* -value < 0.02 calculated using one-sided Students T-test, >2 fold change) for being significantly enriched or depleted when cultures were treated with inhibitor **3**. Dashed lines indicating cutoffs are shown in Figure 6.

	m/z	**Retention Time (min)**	**Fold Change (log2)**	**Significance (-log(P))**	**Asn Labeling**	**Annotation**
**Features Enriched without inhibitor**	557.3657	14.82	-7.264	6.323		
	497.772	14.91	-5.792	5.512		
	423.2214	15.01	-4.649	4.34		
	401.2387	15.1	-4.274	8.034		
	719.3721	10.68	-3.902	2.455		
	309.2064	18.64	-3.799	3.434		
	286.1752	8.05	-3.154	4.685		
	901.4569	18.38	-2.945	4.332		
	615.3674	7.87	-2.731	3.785		
	245.1845	13.03	-2.672	7.17		
	323.2211	20.68	-2.358	5.317		
	925.1407	17.2	-2.309	4.537		
	617.3192	12.29	-2.278	3.548		
	925.8082	17.24	-2.097	4.578		
	295.1645	10.8	-2.04	8.011		
	557.3291	13.94	-1.947	6.22		
	473.2434	11.67	-1.924	2.867		
	217.1526	9.54	-1.869	5.773		
	279.3018	14.01	-1.854	7.238		
	1035.8565	18.29	-1.835	4.359		
	925.4736	17.14	-1.8	5.102		
	620.3097	12.91	-1.773	4.081		
	334.1734	10.12	-1.752	3.653		
	752.8146	16.62	-1.698	4.496		
	648.284	2.91	-1.678	4.541		
	452.2468	9.16	-1.641	2.819		
	606.2605	9.39	-1.639	2.496		
	571.3487	16.75	-1.608	4.958		
	751.3652	8.41	-1.554	2.984		
	567.7716	13.53	-1.531	5.51		
	720.2029	12.49	-1.503	3.627		
	682.347	12.85	-1.493	4.135		
	462.2371	1.26	-1.456	4.852		
	695.8639	14.49	-1.447	3.201		
	864.4332	16.25	-1.433	5.94		
	737.4071	3.1	-1.401	5.206		Edeine A1 [M+H-H2O]
	863.7636	16.14	-1.4	6.094		
	1029.4583	16.09	-1.368	5.33		
	286.6664	8.83	-1.367	2.598		

	864.0977	16.17	-1.352	5.908		
	709.3795	13.85	-1.324	2.3		
	523.2924	17.29	-1.285	6.888		
	557.2968	21.08	-1.241	3.673		
	315.2279	23	-1.234	6.859	Y	*N*-lauroyl-D-Asn [M+H]
	757.0251	13.59	-1.231	5.233		
	659.272	19.82	-1.192	1.969		
	562.2742	9.95	-1.18	1.716		
	818.3999	12.74	-1.104	3.826		
	425.1976	10.43	-1.093	1.959		
	1037.5243	18.16	-1.072	3.623		
	889.8591	17.93	-1.034	5.089		
	316.1577	12.56	-1.029	5.505		
	767.3669	15.46	-1.024	3.751		
	681.0241	14.36	-1.02	2.864		
	554.3166	11.14	-1.016	3.51		
	343.2604	26.33	-1.015	2.278	Y	*N*-myristoyl-D-Asn [M+H]
**Features Enriched with inhibitor**	705.7177	14.07	1.132	2.905		
	763.3999	18.17	1.14	3.551		
	820.3508	20.88	1.286	2.234		
	800.4292	18.4	1.666	2		
	197.0529	1.29	1.675	4.603		
	562.2671	11.06	1.889	4.818		
	569.7702	13.77	2.902	4.077		
	136.0751	3.11	2.921	5.466		
	426.2381	17.85	2.954	3.558		
	1155.5952	12.32	3.249	2.475	Y	pre(myristoyl)Edeine A1 [M+2K-H]
	392.2535	17.48	3.415	4.528		
	326.668	9.74	3.74	2.317		
	618.3254	12.97	4.159	5.733		
	207.1119	12.28	4.628	6.086		
	1027.5472	12.74	4.68	6.722	Y	
	765.3966	15.66	5.432	7.875		
	344.2418	12.94	6.124	5.261		
	374.6883	15.68	6.128	5.948		
	1069.3861	15.69	6.474	7.626	Y	
	383.2006	15.66	6.807	6.053		
	1039.0947	16.24	9.467	8.193	Y	
	493.3028	14.68	11.27	7.273		

Synthetic procedures

All solvents for synthesis were obtained from Millipore-Sigma unless otherwise noted. All NMR solvents were purchased from Cambridge Isotope Laboratories (Tewksbury, MA). NMR chemical shifts are reported in parts per million downfield from tetramethylsilane using the solvent resonance as internal standard for ^{1}H ($CDCl_3$ = 7.26 ppm, DMSO-d_6 = 2.50 ppm, CD_2Cl_2 = 5.32 ppm) and ^{13}C ($CDCl_3$ = 77.25 ppm, DMSO-d_6 = 39.52 ppm, CD_2Cl_2 = 54 ppm). Data are reported as follows: chemical shift, integration multiplicity (s = singlet, d = doublet, t = triplet, q = quartet, quint = quintet, m = multiplet), coupling constant, integration, and assignment. NMR spectra were collected in the Magnetic Resonance Laboratory in Harvard University Department of Chemistry and Chemical Biology and visualized and processed using MestreNova, version 11.0.2-18153 (Mestrelab Research S.L., Escondido, CA). High-resolution LC-MS (HRMS) analyses of synthetic compounds were performed on an Agilent 6530 Q-TOF Mass Spectrometer fitted with a dual-spray electrospray ionization (ESI) source. The capillary voltage was set to 3.5 kV, the fragmentor voltage to 175 V, the skimmer voltage to 65 V, and the Oct 1 RF to 750 V. The drying gas temperature was maintained at 275 °C with a flow rate of 8 L/min and a nebulizer pressure of 35 psi. A standard calibrant mix was introduced continuously during all experiments via the dual-spray ESI source. Low-resolution mass spectrometry analysis (LRMS) was conducted by direct infusion on an Advion CMS single-quadrupole mass spectrometer in ESI+ mode.

General Procedure A

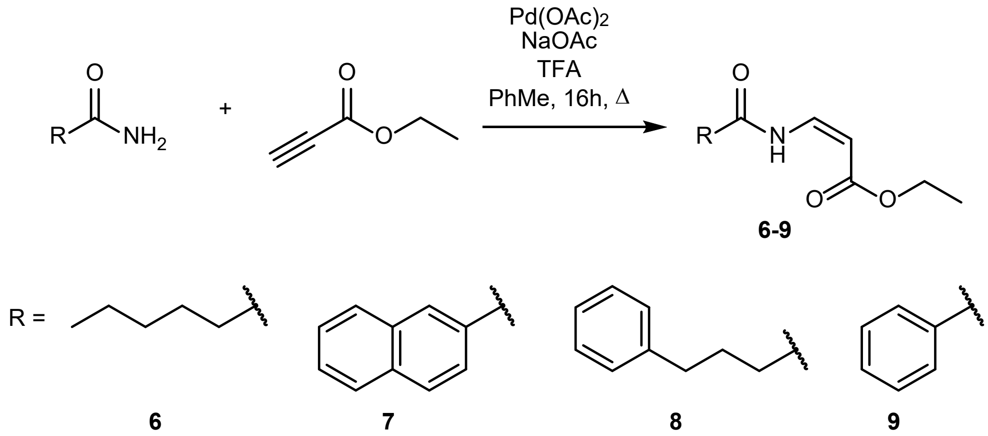

Intermediates **6**, **7**, **8**, and **9** were prepared using the procedure described by López and coworkers.[2] Briefly, an oven-dried glass microwave vial was charged with palladium (II) acetate (3.3 mg, 0.015 mmol, 0.01 equiv), sodium acetate (246 mg, 3 mmol, 2.0 equiv,), the corresponding amide (1.5 mmol, 1.0 equiv), and anhydrous toluene (3.75 mL, 0.4 M). Trifluoroacetic acid (574 µL, 7.5 mmol, 5.0 equiv) was added and the reaction mixture was stirred for 5 minutes under nitrogen atmosphere at room temperature, and then ethyl propiolate (228 µL, 2.25 mmol, 1.5 equiv) was added dropwise. The reaction mixture was then stirred for 5 min and then heated at 80 °C overnight. The reaction mixture was diluted with EtOAc and water was added. The organic layer was separated, and the aqueous layer was extracted with EtOAc

three times. The combined organic layers were washed with brine, dried over Na_2SO_4, and concentrated *in vacuo*. The residue was dissolved in EtOAc and purified by flash chromatography on silica (0-100% EtOAc in hexanes). In some cases, when left in solution for extended periods of time, these compounds were observed to equilibrate between the *cis* and *trans* isomers. In cases where this was observed, the chromatography was repeated under the same conditions to separate the isomers and use only the *cis* isomer as shown above in the subsequent step. Yields below refer to the final isolated amount of the *cis* isomer.

6: Yield: 137 mg (81%, reaction performed on 1 equiv = 0.4 mmol scale). ^{1}H NMR (400 MHz DMSO-d$_6$): δ (ppm) = 10.62 (d, J = 11.2 Hz, 1H), 7.81 (dd, J = 14.2, 11.2 Hz, 1H), 5.42 (d, J = 14.1 Hz, 1H), 4.08 (q, J = 7.1 Hz, 2H), 2.26 (t, J = 7.4 Hz, 2H), 1.54 (quint, J = 7.4 Hz, 2H), 1.34 – 1.16 (m, 7H), 0.90 – 0.81 (m, 3H). ^{13}C NMR (101 MHz, DMSO-d$_6$) δ = 171.9, 168.4, 138.5, 95.9, 60.2, 35.9, 31.2, 24.6, 22.8, 14.7, 14.3. LRMS (ESI): calcd for $C_{11}H_{20}NO_3$ $[M+H]^+$, *m/z* 214.14; found, *m/z* 214.15.

7: Yield: 165 mg (41%). ^{1}H NMR (400 MHz DMSO-d$_6$): δ (ppm) = 11.46 (d, J = 11.1 Hz, 1H), 8.54 (s, 1H), 8.20 – 8.10 (m, 2H), 8.05 (d, J = 8.0 Hz, 1H), 7.91 (d, J = 8.6 Hz, 1H), 7.78 (t, J = 10.6 Hz, 1H), 7.73 – 7.63 (m, 2H), 5.36 (d, J = 8.9 Hz, 1H), 4.22 (q, J = 7.1 Hz, 2H), 1.28 (t, J = 7.1 Hz, 3H). ^{13}C NMR (101 MHz, DMSO-d$_6$) δ = 168.7, 163.8, 138.8, 134.9, 132.1, 129.3, 129.1, 129.0, 128.7, 128.5, 127.8, 127.3, 123.2, 97.2, 60.1, 14.1. LRMS (ESI): calcd for $C_{16}H_{16}NO_3$ $[M+H]^+$, *m/z* 270.11; found, *m/z* 270.11.

8: Yield: 121 mg (31%). ^{1}H NMR (400 MHz DMSO-d$_6$): δ (ppm) = δ 10.34 (d, J = 11.6 Hz, 1H), 7.45 (dd, J = 11.6, 9.0 Hz, 1H), 7.32-7.24 (m, 2H), 7.22-7.14 (m, 3H), 5.11 (d, J = 9.0 Hz, 1H), 4.13 (q, J = 7.1 Hz, 2H), 2.59 (dd, J = 8.7, 6.7 Hz, 2H), 2.45 (t, J = 7.4 Hz, 2H), 1.85 (quint, J = 7.5 Hz, 2H), 1.21 (t, J = 7.1 Hz, 3H). ^{13}C NMR (101 MHz, DMSO-d$_6$) δ = 171.1, 167.8, 141.4, 128.4, 128.3, 126.1, 125.9, 95.4, 84.2, 59.6, 34.4, 26.2, 14.1. LRMS (ESI): calcd for $C_{15}H_{20}NO_3$ $[M+H]^+$, *m/z* 262.14; found, *m/z* 262.12 .

9: Yield: 110 mg (33%). ^{1}H NMR (400 MHz DMSO-d$_6$): δ (ppm) = 11.37 (d, J = 11.1 Hz, 1H), 7.89 (dd, J = 7.5, 1.7 Hz, 2H), 7.77 – 7.67 (m, 2H), 7.61 (dd, J = 8.3, 6.8 Hz, 2H), 5.34 (d, J = 8.8 Hz, 1H), 4.20 (q, J = 7.1 Hz, 2H), 1.26 (t, J = 7.1 Hz, 3H). ^{13}C NMR (101 MHz, DMSO-d$_6$) δ = 168.8, 163.6, 138.8, 133.2, 131.8, 129.2, 127.3, 97.1, 60.1, 14.1. LRMS (ESI): calcd for $C_{12}H_{14}NO_3$ $[M+H]^+$, *m/z* 220.10; found, *m/z* 220.12 .

General Procedure B

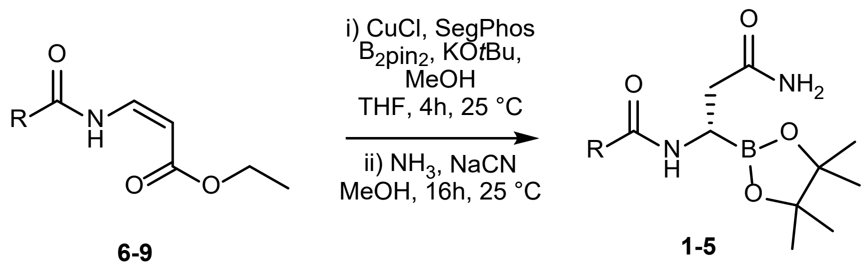

An oven-dried glass microwave vial or round bottom flask was charged with CuCl (2 mg, 0.02 mmol, 0.1 equiv), B_2pin_2 (56 mg, 0.22 mmol, 1.1 equiv), and SegPhos (13 mg, 0.022 mmol, 0.11 equiv. (*S*)-SegPhos was used for the preparation of MRV03-037 (**1**), MRV03-068 (**2**), MRV03-069 (**3**), and MRV03-070 (**4**); (*R*)-SegPhos was used for the preparation of MRV03-095 (**5**). The vial was evacuated and backfilled with argon three times. Anhydrous THF (0.5 mL) was added followed by KO*t*Bu (650 μL, 1 M solution in THF) and the mixture was stirred for 30 minutes at room temperature. A solution of the corresponding intermediate (**6-9**) in THF was added (1 mL of a 0.2 M solution, 0.2 mmol, 1 equiv), followed by MeOH (32 μL, 0.8 mmol, 4 equiv), and the reaction was stirred for 4 hours at room temperature. The reaction was then concentrated *in vacuo*. The residue was taken up in 3:1 hexanes:EtOAc and filterd over a short plug of deactivated (35 wt% H_2O) silica. The filtrate was concentrated *in vacuo* in a round bottom flask with a stir bar. NaCN (2 mg, 0.04 mmol, 0.2 equiv) was added, followed by a solution of NH_3 in MeOH (7 M, 6 mL). The mixture was stirred at room temperature for 16 hours and then concentrated *in vacuo* and purified by flash chromatography using deactivated silica (35 wt% H_2O) and eluting with 0-20% MeOH in EtOAc.

MRV03-037 (**1**): Yield: 31.2 mg (50% over two steps) ^1H NMR (400 MHz CD_2Cl_2): δ (ppm) = 9.02 (s, 1H), 7.53 (s, 1H), 5.78 (s, 1H), 2.76 (t, J = 6.3 Hz, 1H), 2.49 – 2.36 (m, 2H), 2.32 (d, J = 7.6 Hz, 2H), 1.58 (q, J = 7.5 Hz, 2H), 1.35 – 1.22 (m, 4H), 1.19 – 1.09 (m, 12H), 0.92 – 0.81 (m, 3H).j ^{13}C NMR (101 MHz, CD_2Cl_2) δ (ppm) = 179.5, 176.7, 80.7, 42.7 (br)*, 37.5, 31.7, 31.6, 25.6, 25.4, 25.3, 22.7, 14.2. ^{11}B NMR (128 MHz, CD_2Cl_2) δ (ppm) = 14.9 (br s). HRMS (ESI): calcd for $C_{15}H_{30}BN_2O_4$ $[M+H]^+$, *m/z* 313.2299; found, *m/z* 313.2230.

*the broad peak at 42.7 ppm in the ^{13}C NMR spectrum of **1** corresponds to the carbon which is bound directly to the boron atom. Due to the line broadening effect of the quadrupolar boron nucleus, this signal is only visible after a very large number of scans and is not visible is the ^{13}C NMR spectra of the other compounds reported here.

MRV03-068 (**2**): Yield: 11 mg (15% over two steps) ^1H NMR (400 MHz $CDCl_3$): δ (ppm) = 9.15 (s, 1H), 8.44 (s, 1H), 7.92 – 7.77 (m, 4H), 7.62 – 7.46 (m, 2H), 6.91 (s, 1H), 5.55 (s, 1H), 3.20 – 3.13 (m, 1H), 2.75 – 2.57 (m, 2H), 1.28 (m, 12H). ^{13}C NMR (101 MHz, $CDCl_3$) δ (ppm) = 176.5, 171.9, 135.8, 132.3, 130.4, 129.4, 128.9, 128.8, 127.9, 127.2, 123.6, 123.4, 80.6, 36.7, 25.5, 25.2. ^{11}B NMR (128 MHz, $CDCl_3$) δ (ppm) = 14.0 (br s). HRMS (ESI): calcd for $C_{20}H_{26}BN_2O_4$ $[M+H]^+$, *m/z* 369.1986; found, *m/z* 369.1987.

MRV03-069 (**3**): Yield: 9.5 mg (13% over two steps) ^1H NMR (400 MHz CD_2Cl_2): δ (ppm) = 8.57 (s, 1H), 7.32 – 7.23 (m, 2H), 7.23 – 7.13 (m, 4H), 5.54 (s, 1H), 2.81 (t, J = 6.4 Hz, 1H), 2.68 – 2.61 (m, 2H), 2.53 – 2.39 (m, 2H), 2.34 (t, J = 7.6 Hz, 2H), 1.97 – 1.82 (m, 2H), 1.20 – 1.11 (m, 12H). ^{13}C NMR (101 MHz, CD_2Cl_2) δ (ppm) = 178.5, 176.2, 141.1, 128.6, 128.5, 126.2, 80.4, 36.8, 34.9, 30.6, 26.7, 25.1, 24.9. ^{11}B NMR (128 MHz, CD_2Cl_2) δ (ppm) = 15.4 (br s). HRMS (ESI): calcd for $C_{19}H_{30}BN_2O_4$ $[M+H]^+$, *m/z* 361.2299; found, *m/z* 361.2296.

MRV03-070 (**4**): Yield: 10 mg (16 % over two steps) ^1H NMR (400 MHz $CDCl_3$): δ (ppm) = δ 8.84 (s, 1H), 7.89 – 7.82 (m, 2H), 7.61 – 7.52 (m, 1H), 7.48 – 7.38 (m, 2H), 6.67 (s, 1H), 5.46 (s, 1H), 3.14-3.06 (m, 1H), 2.70 – 2.51 (m, 2H), 1.24 (m, 12H). ^{13}C NMR (101 MHz, $CDCl_3$) δ

(ppm) = 176.4, 171.8, 133.9, 128.9, 128.4, 126.7, 80.6, 36.5, 25.4, 25.1. ^{11}B NMR (128 MHz, $CDCl_3$) δ (ppm) = 16.2 (br s). HRMS (ESI): calcd for $C_{16}H_{24}BN_2O_4$ $[M+H]^+$, *m/z* 319.1829; found, *m/z* 319.1832.

MRV03-095 (**5**): Yield: 12.3 mg (17% over two steps) ^{1}H NMR (400 MHz CD_2Cl_2): δ (ppm) = 8.48 (s, 1H), 7.36 – 7.23 (m, 2H), 7.23 – 7.13 (m, 3H), 7.02 (s, 1H), 5.48 (s, 1H), 2.81 (t, J = 6.2 Hz, 1H), 2.67 – 2.61 (m, 3H), 2.52 – 2.38 (m, 2H), 2.38 – 2.27 (m, 2H), 2.00 – 1.88 (m, 2H), 1.22 – 1.12 (m, 12H). ^{13}C NMR (101 MHz, CD_2Cl_2) δ (ppm) = 178.5, 176.1, 141.2, 128.7, 128.6, 126.2, 80.4, 36.8, 34.9, 30.8, 26.8, 25.2, 24.9. ^{11}B NMR (128 MHz, CD_2Cl_2) δ (ppm) = 15.6 (br s). HRMS (ESI): calcd for $C_{19}H_{30}BN_2O_4$ $[M+H]^+$, *m/z* 361.2299; found, 361.2298 *m/z*.

NMR Spectra

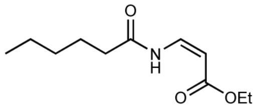

Compound **6**: ^{1}H NMR (400 MHz DMSO-d$_6$)

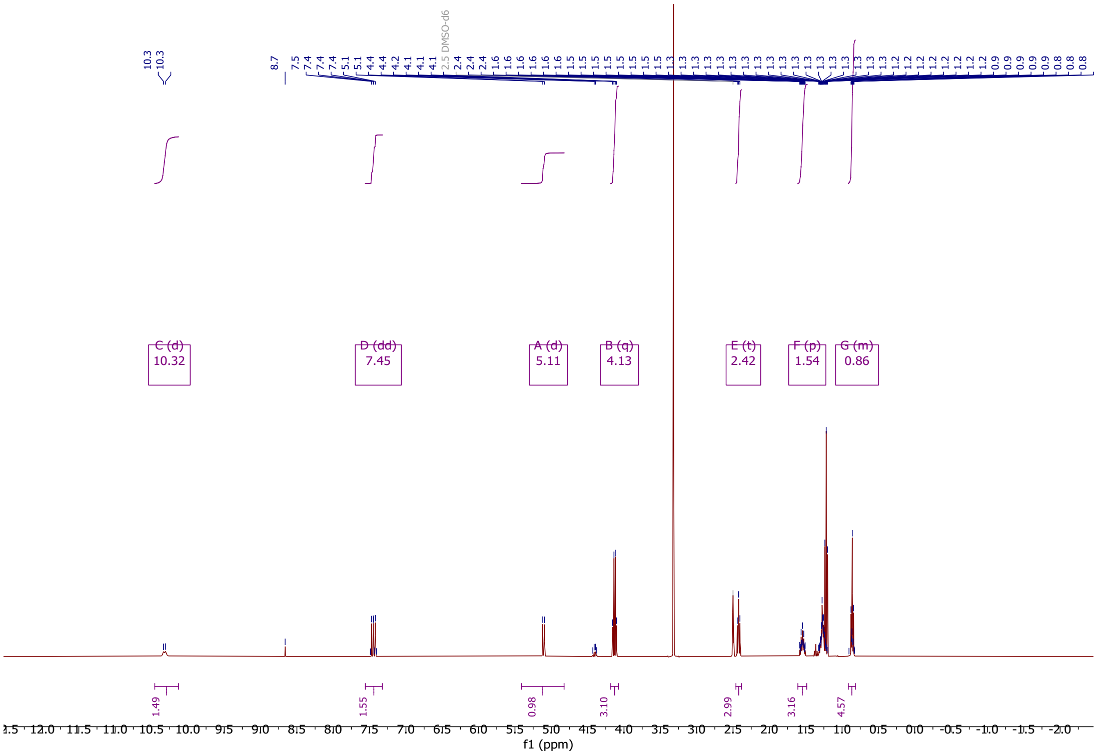

^{13}C NMR (101 MHz, DMSO-d$_6$)

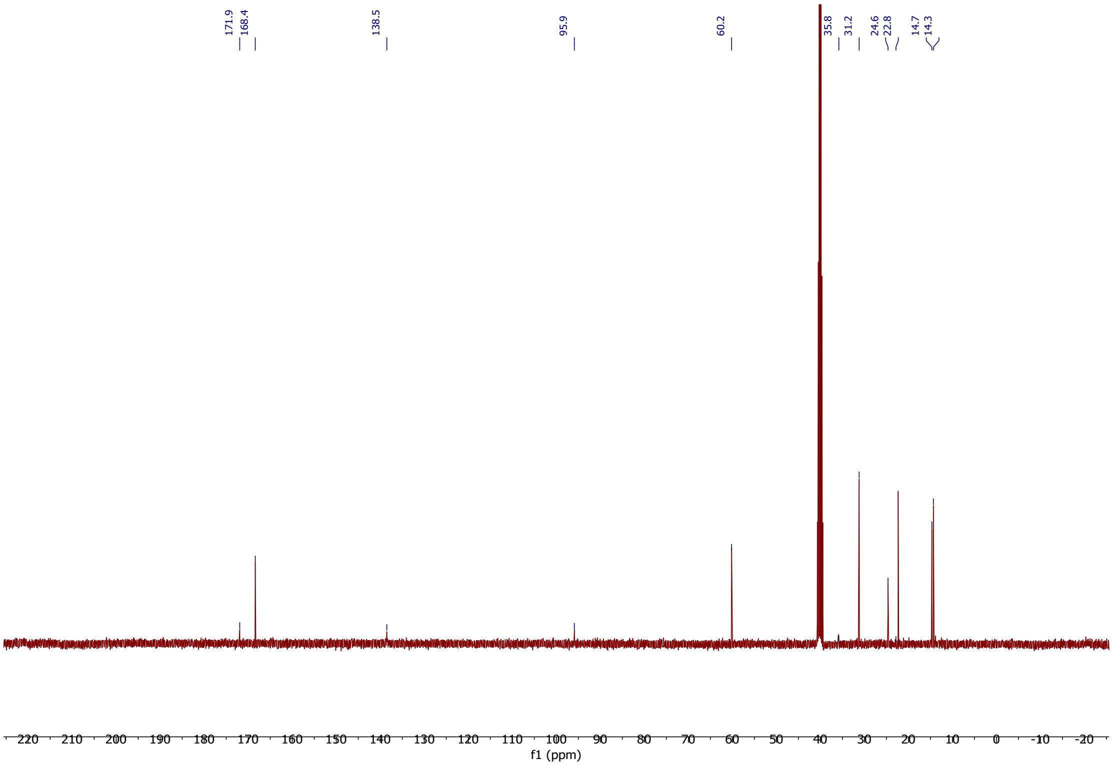

Compound **7**: ^{1}H NMR (400 MHz DMSO-d$_6$)

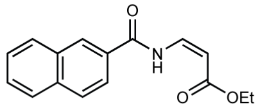

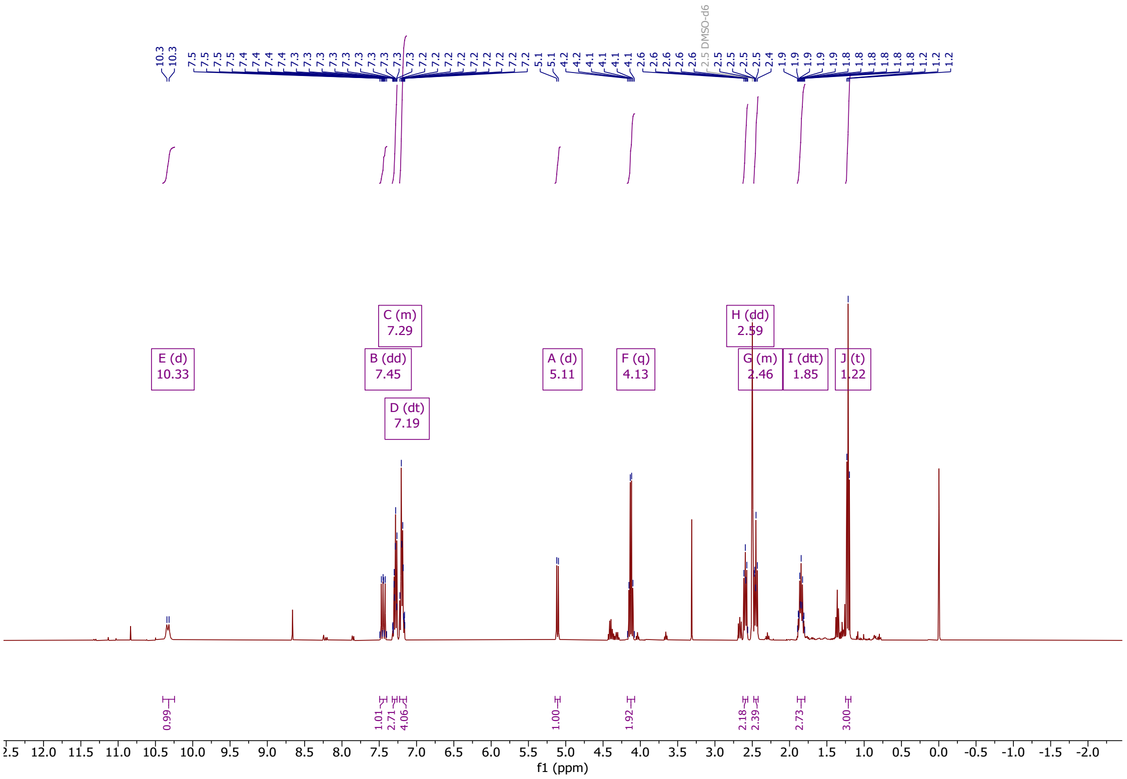

^{13}C NMR (101 MHz, DMSO-d$_6$)

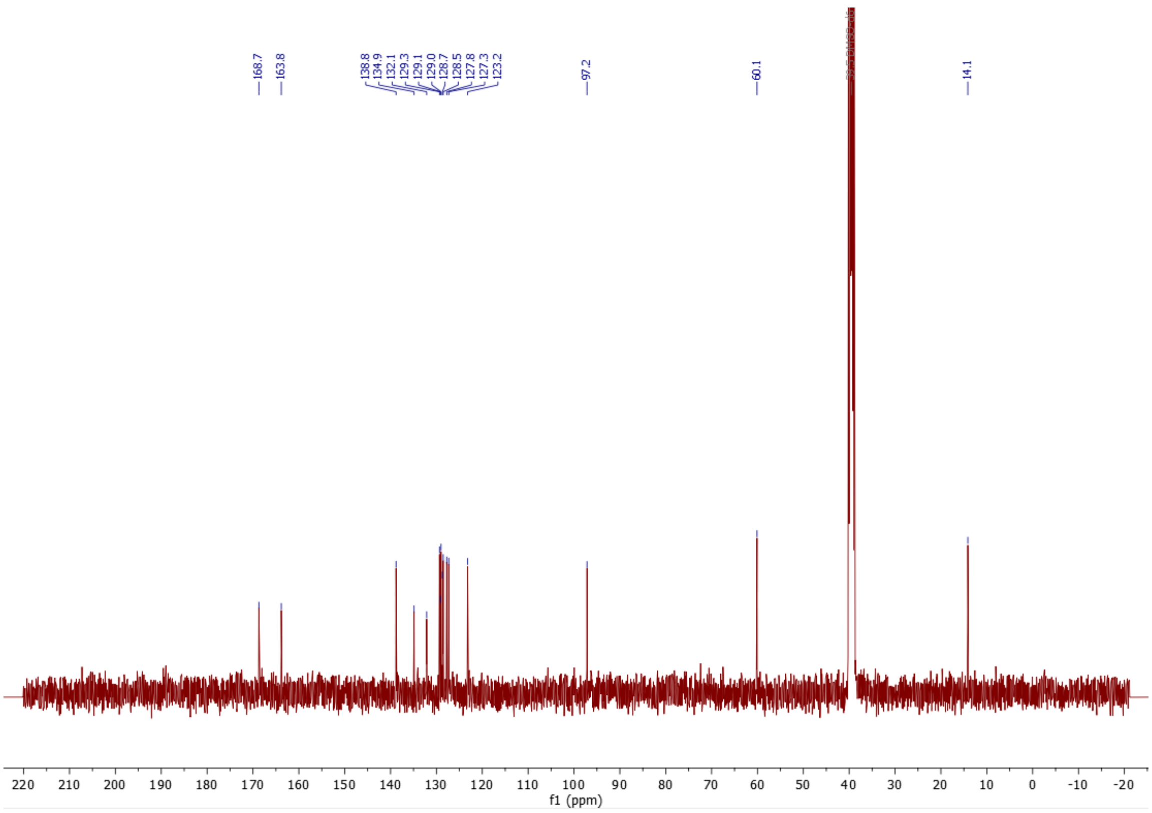

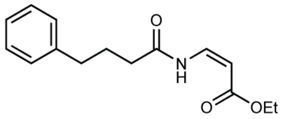

Compound **8**: ^{1}H NMR (400 MHz DMSO-d_6)

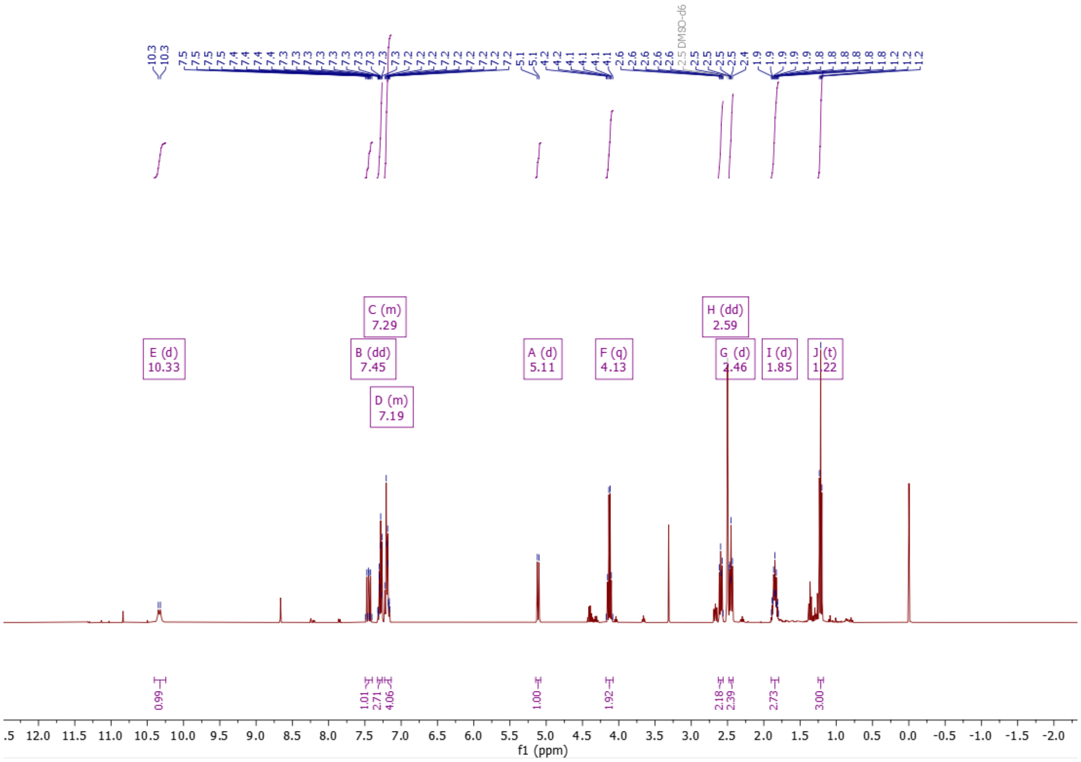

^{13}C NMR (101 MHz, DMSO-d_6)

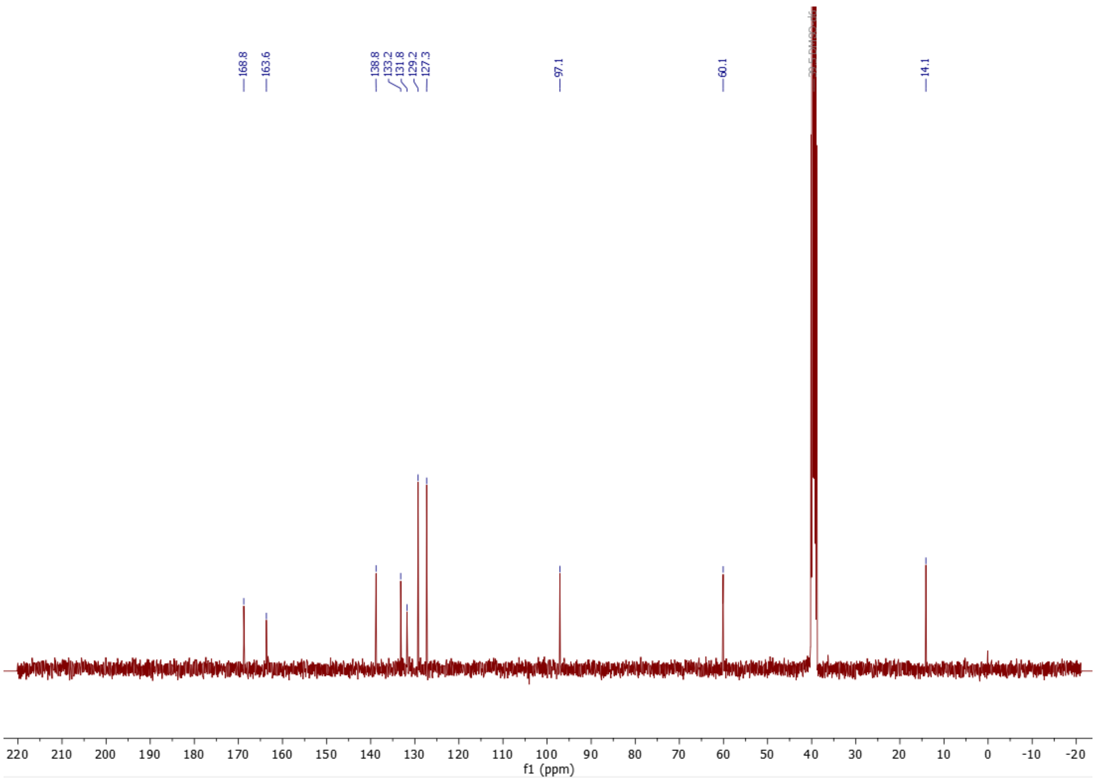

Compound **9**: ^{1}H NMR (400 MHz DMSO-d$_6$)

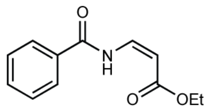

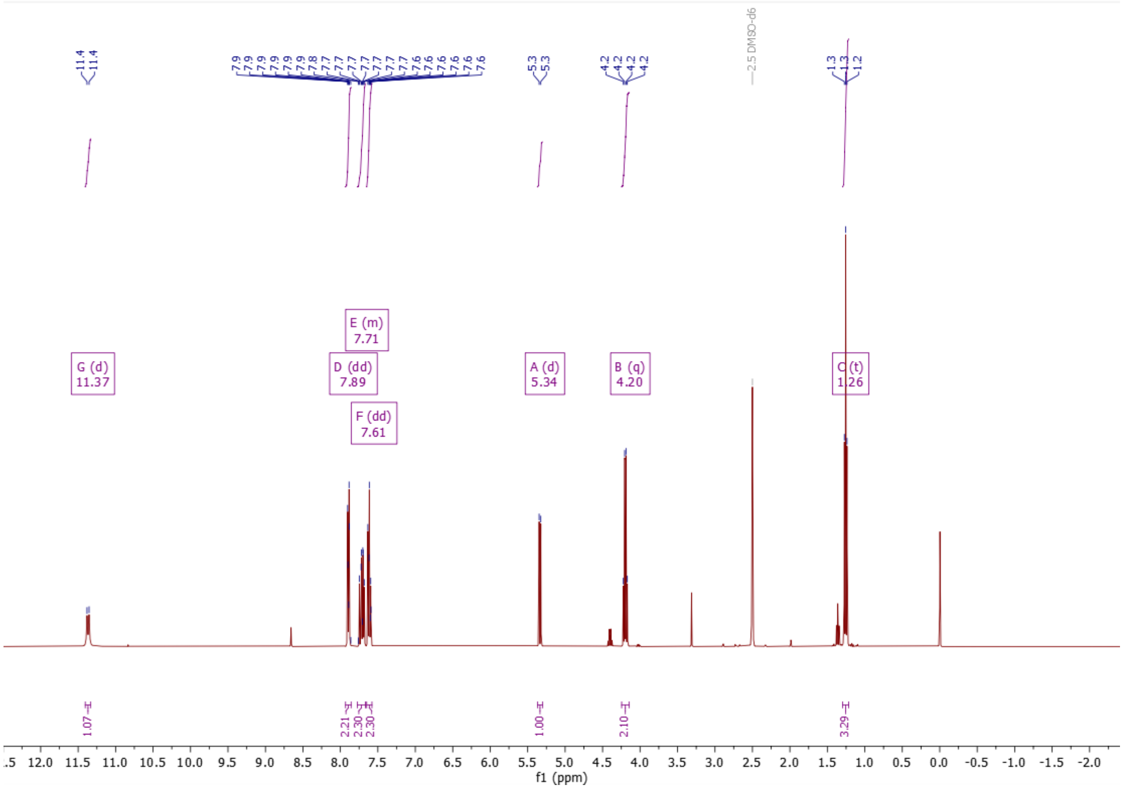

^{13}C NMR (101 MHz, DMSO-d$_6$)

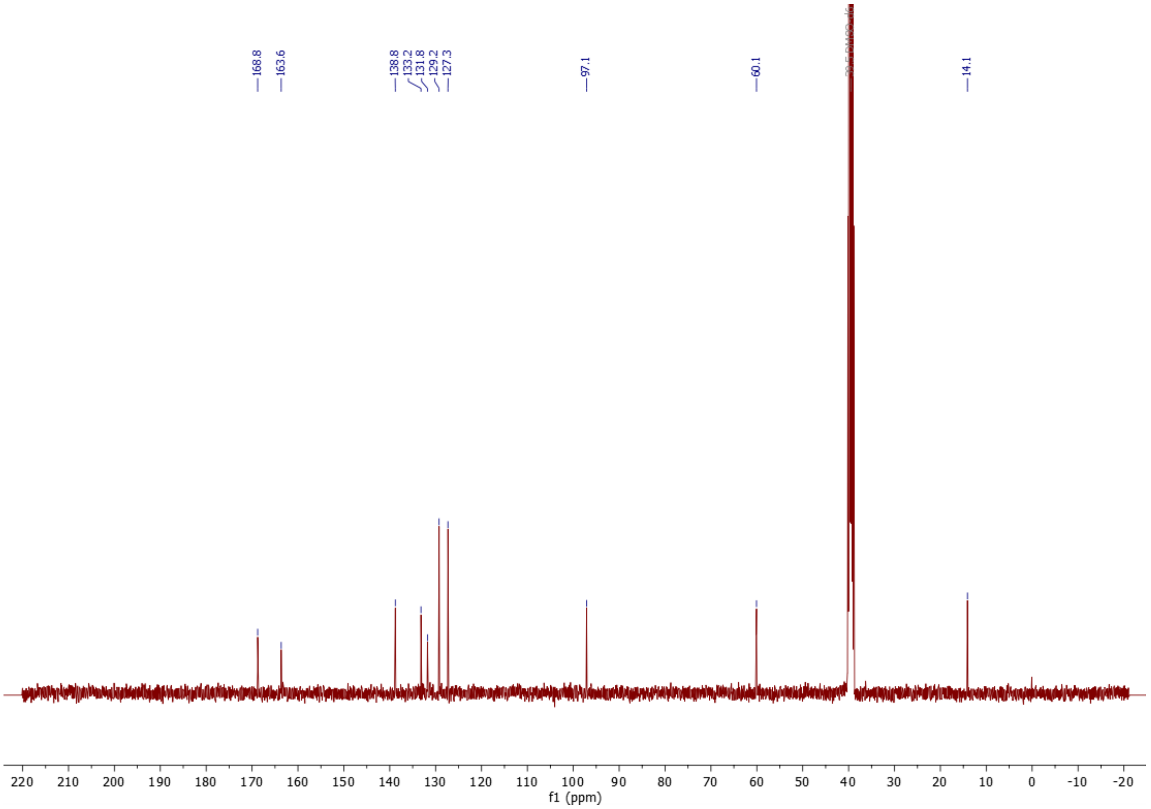

Compound **1**: ^{1}H NMR (400 MHz CD_2Cl_2)

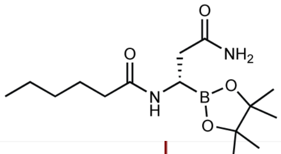

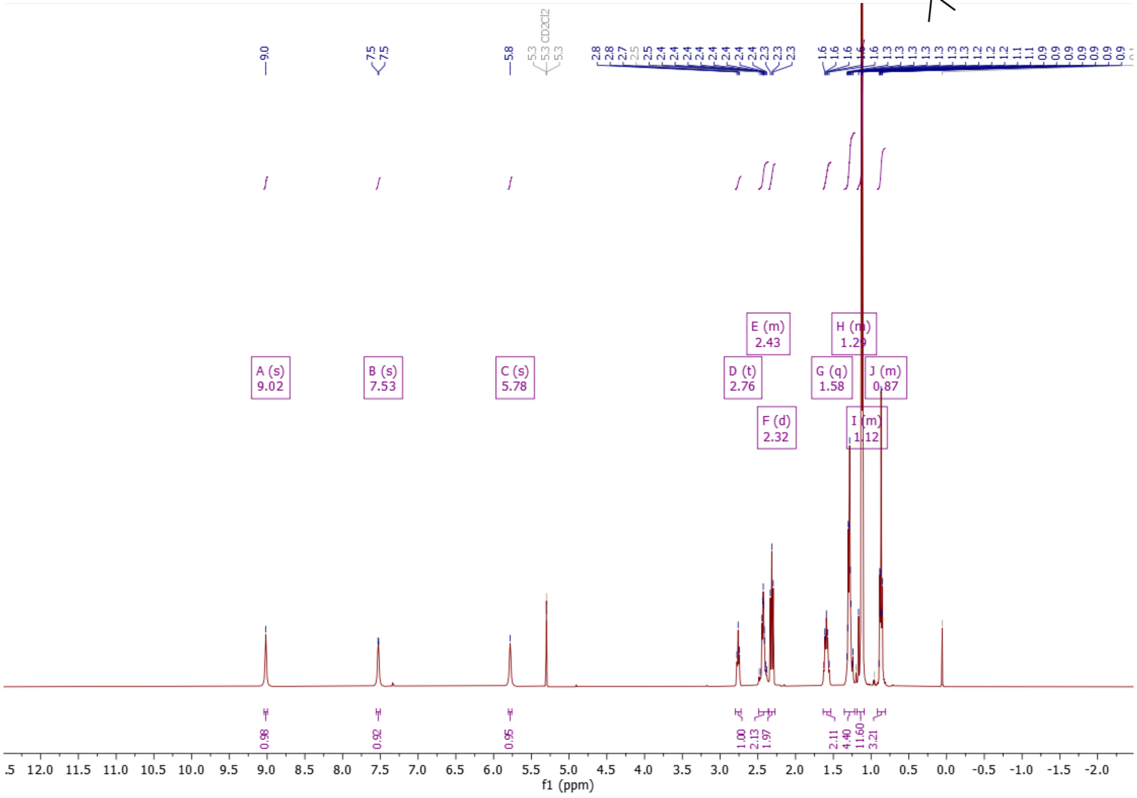

^{13}C NMR (101 MHz, CD_2Cl_2)

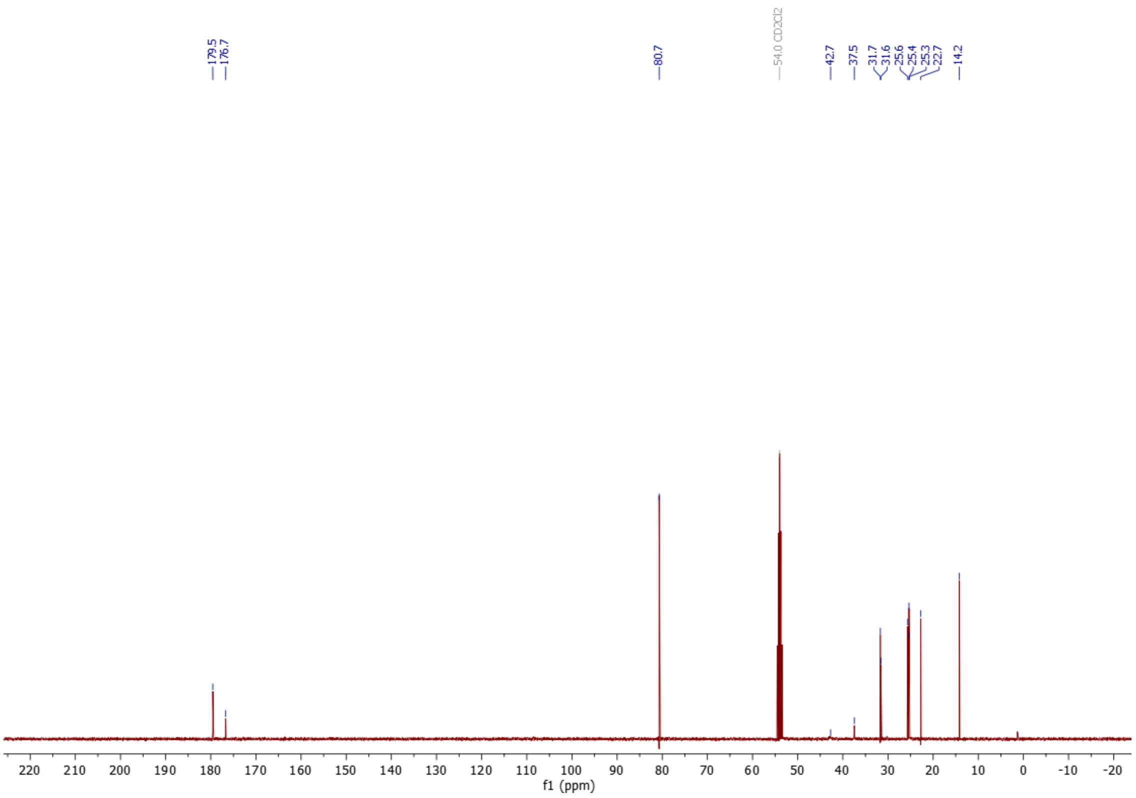

^{11}B NMR (128 MHz, CD_2Cl_2) (borosilicate glass tube)

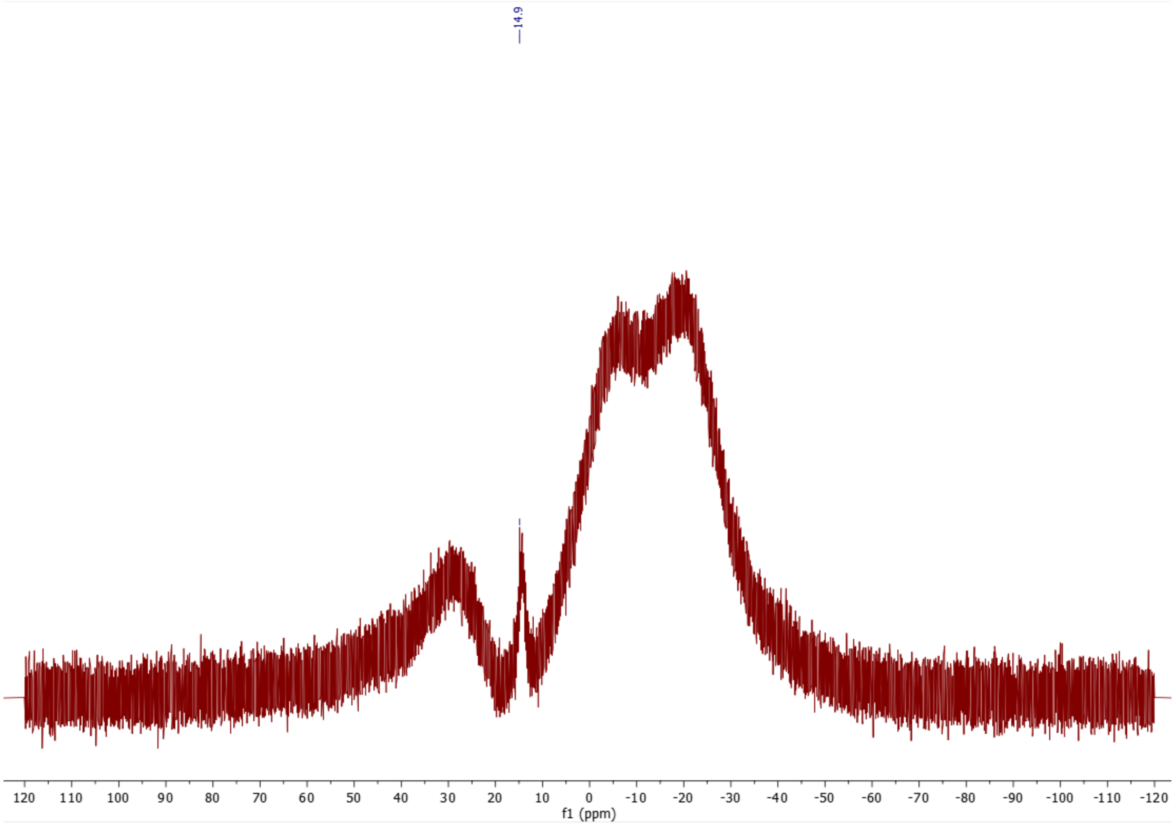

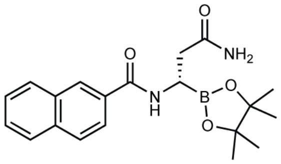

Compound **2**: ^{1}H NMR (400 MHz CDCl3)

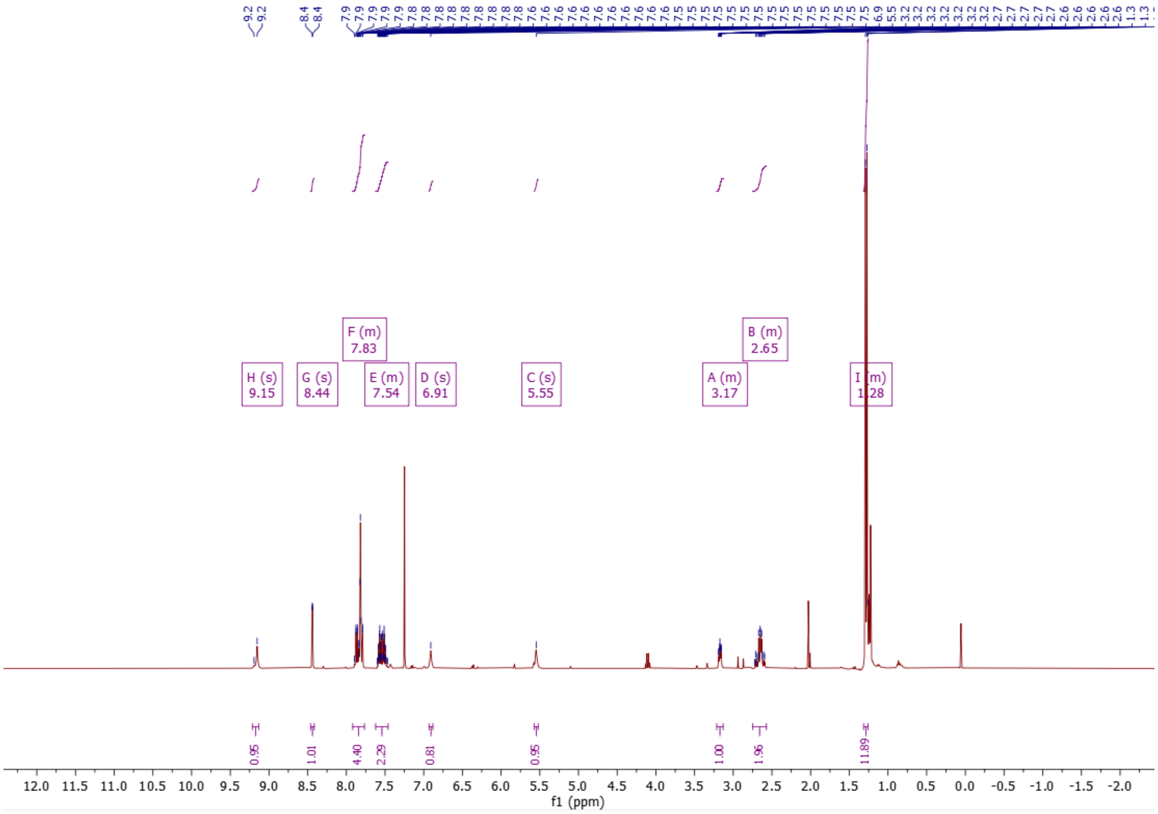

^{13}C NMR (101 MHz, CDCl3)

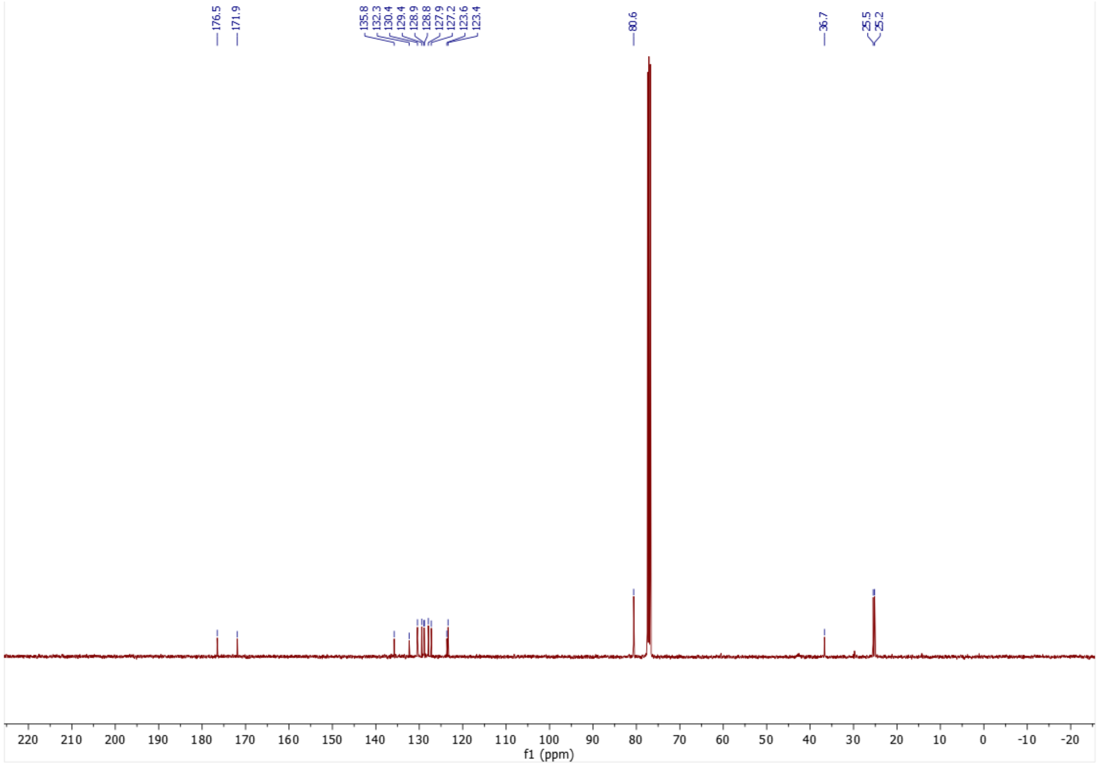

^{11}B NMR (128 MHz, $CDCl_3$) (quartz tube)

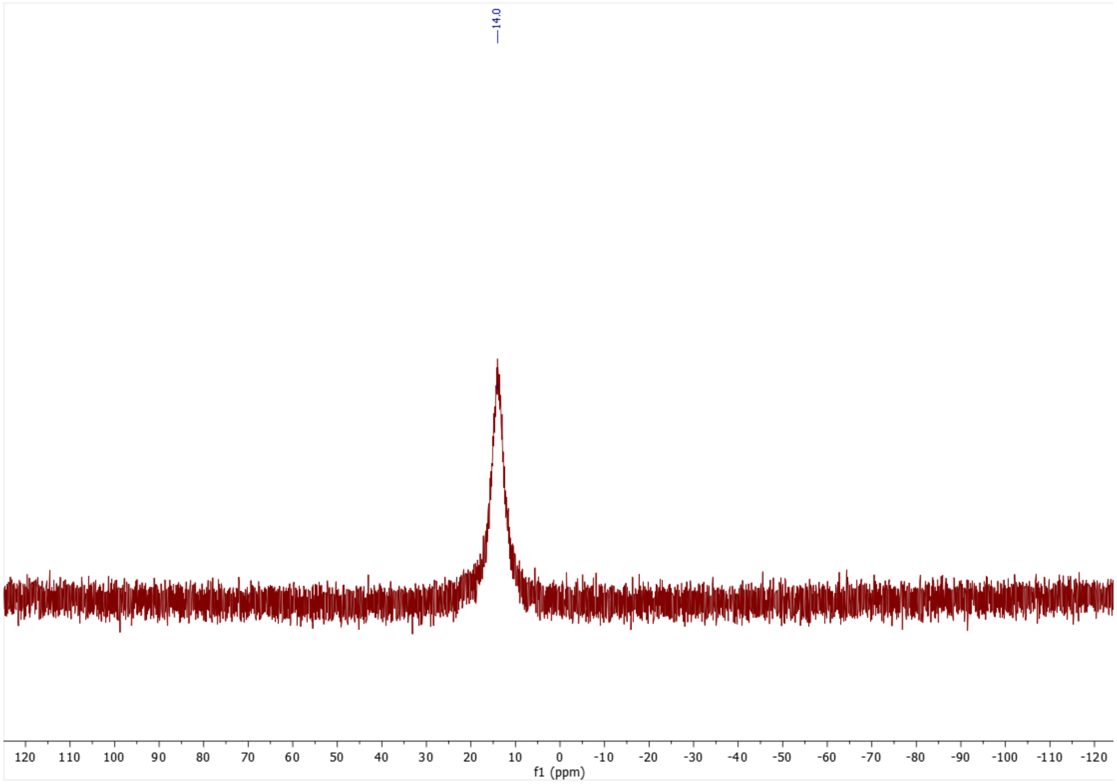

Compound **3**: ^{1}H NMR (400 MHz, CD_2Cl_2)

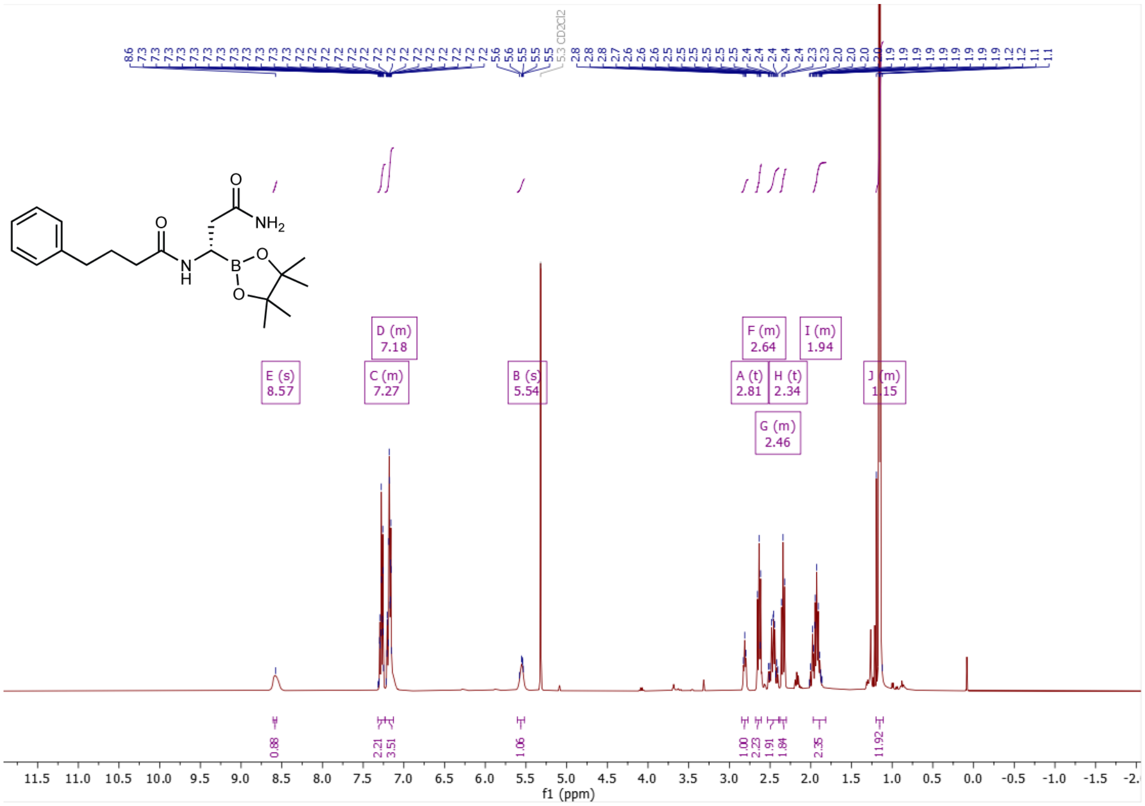

^{13}C NMR (101 MHz, CD_2Cl_2)

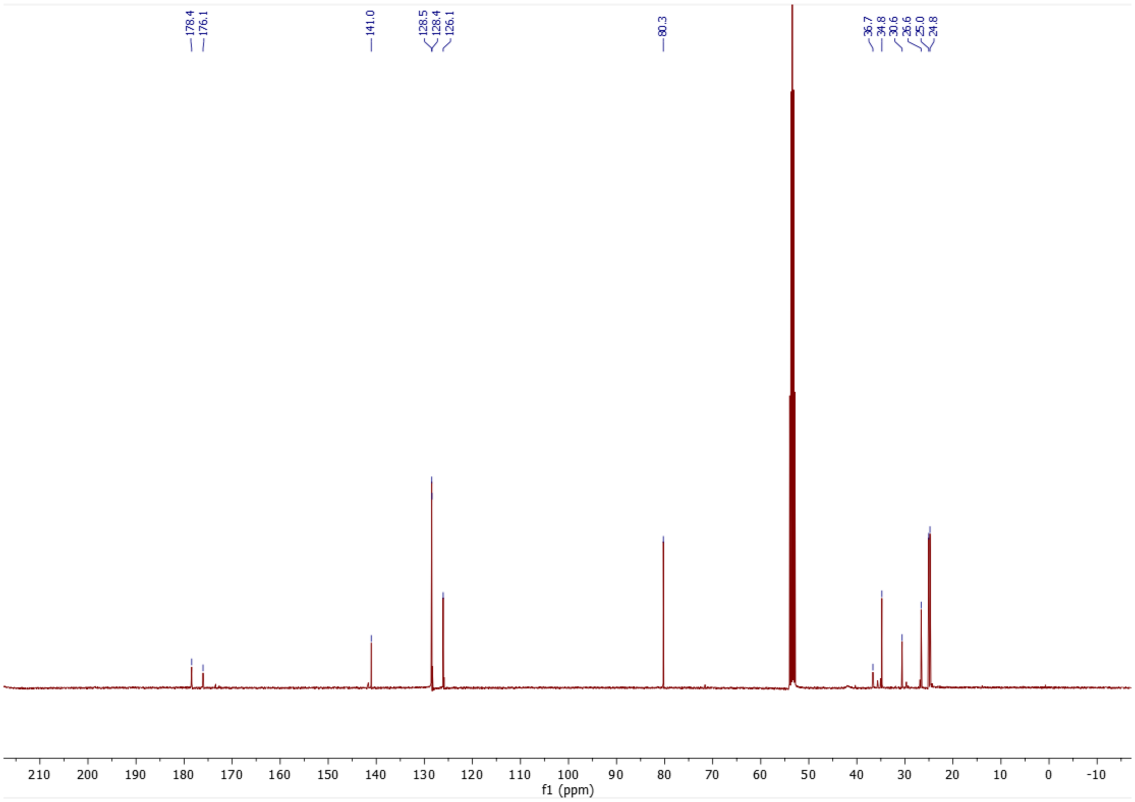

^{11}B NMR (128 MHz, CD_2Cl_2) (quartz tube)

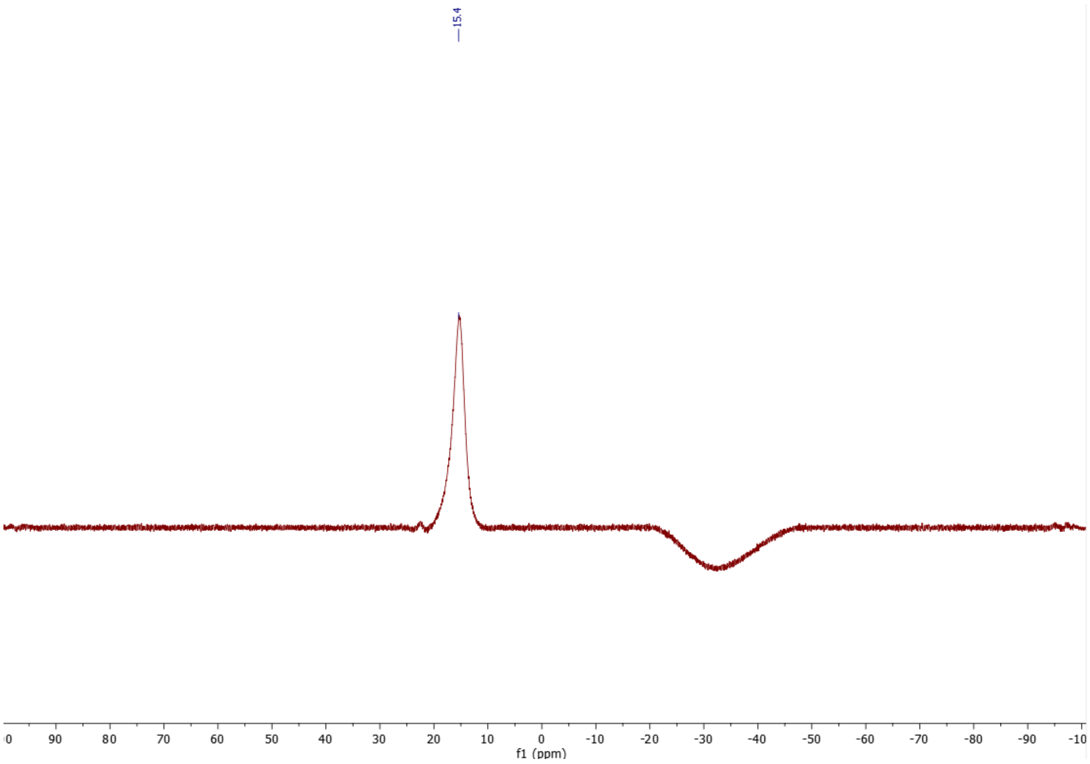

Compound **5**: ^{1}H NMR (400 MHz CD_2Cl_2)

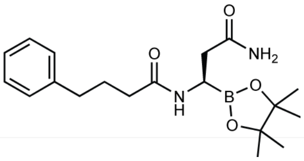

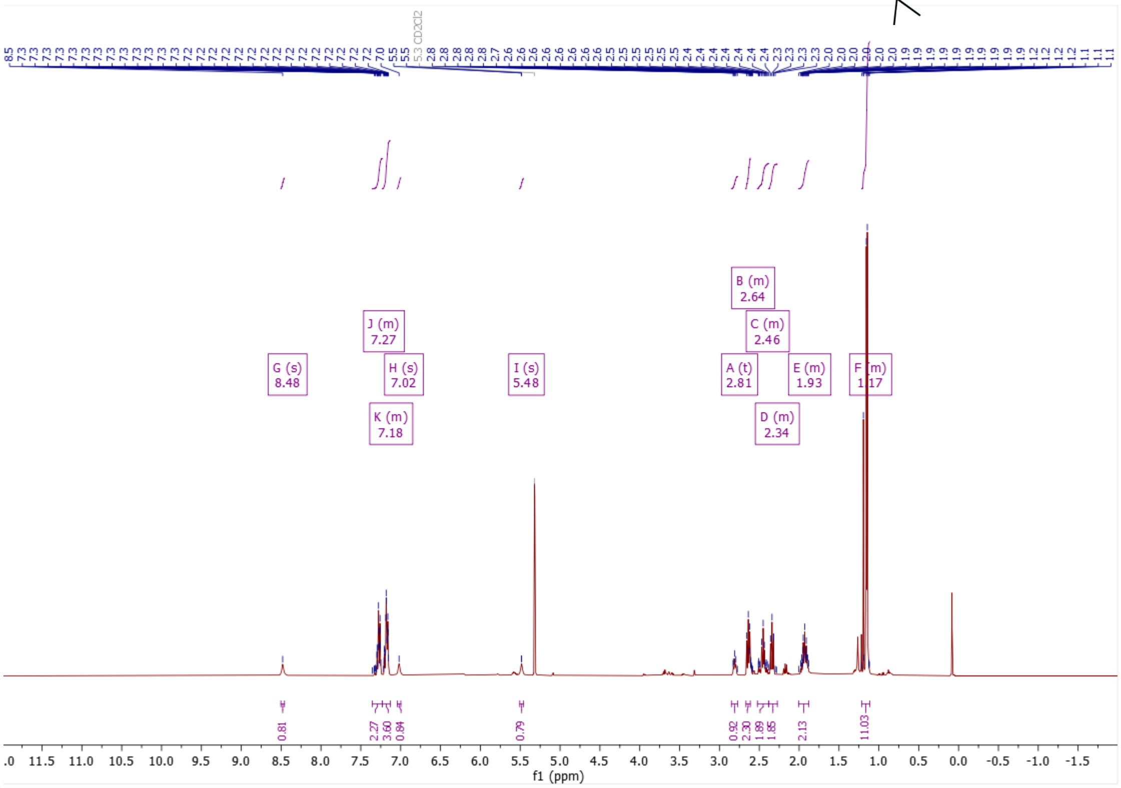

^{13}C NMR (101 MHz, CD_2Cl_2)

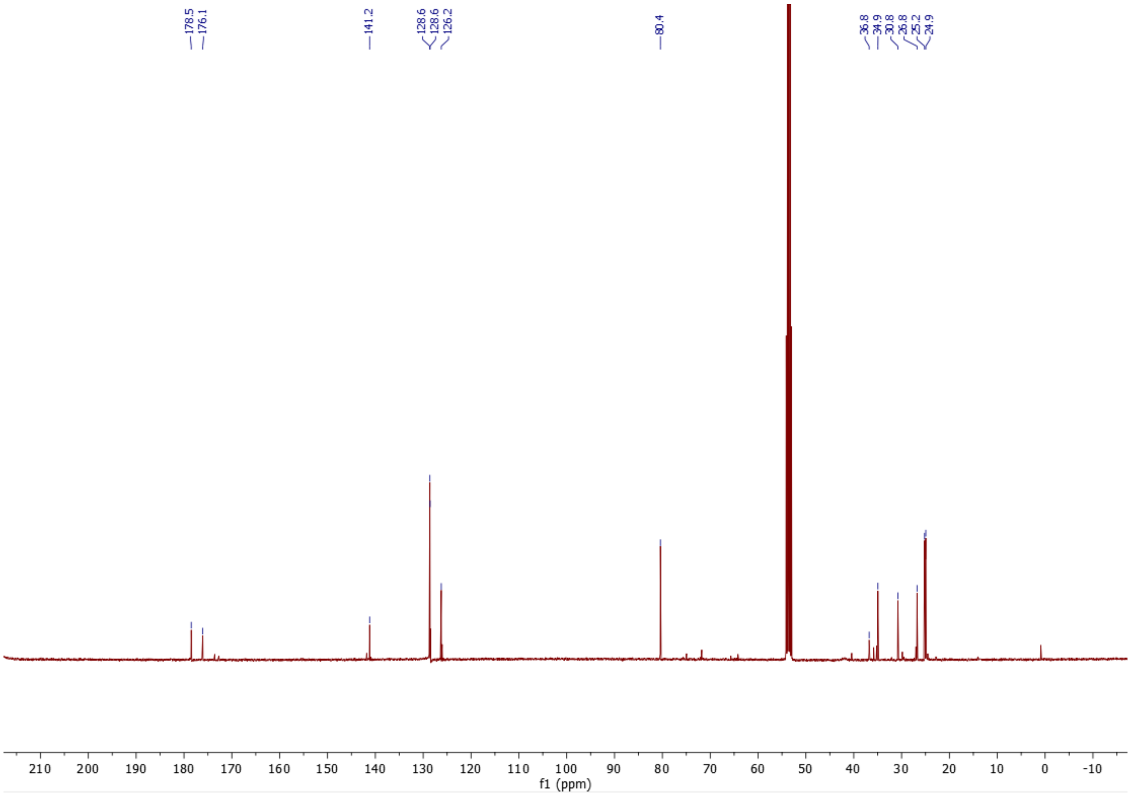

^{11}B NMR (128 MHz, CD_2Cl_2) (quartz tube)

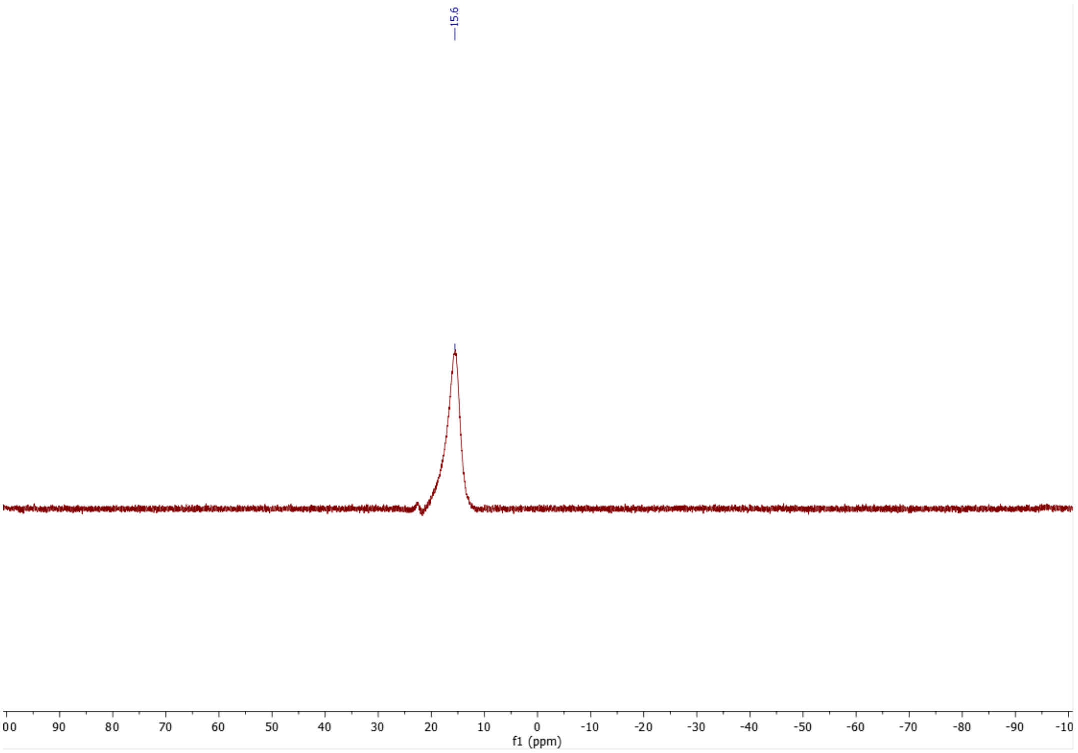

Compound **4**: ^{1}H NMR (400 MHz $CDCl_3$)

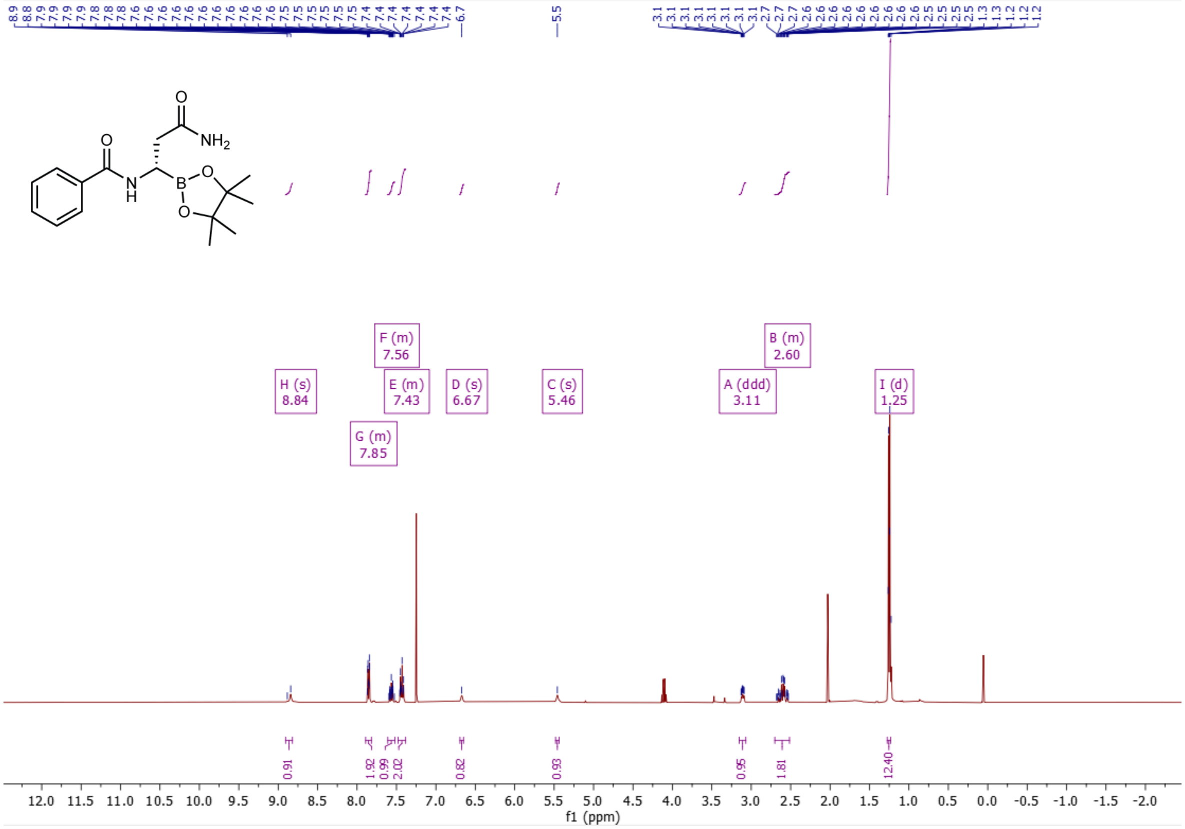

^{13}C NMR (101 MHz, $CDCl_3$)

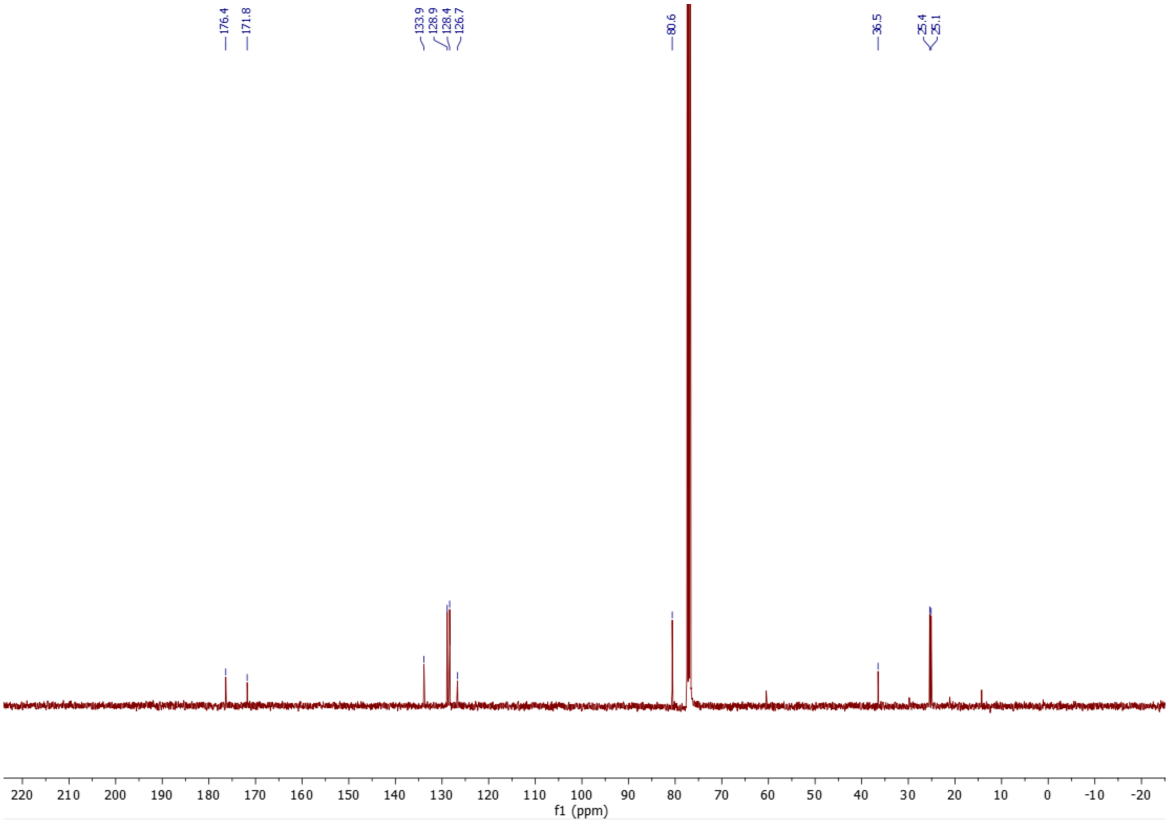

^{11}B NMR (128 MHz, CD_2Cl_2) (quartz tube)

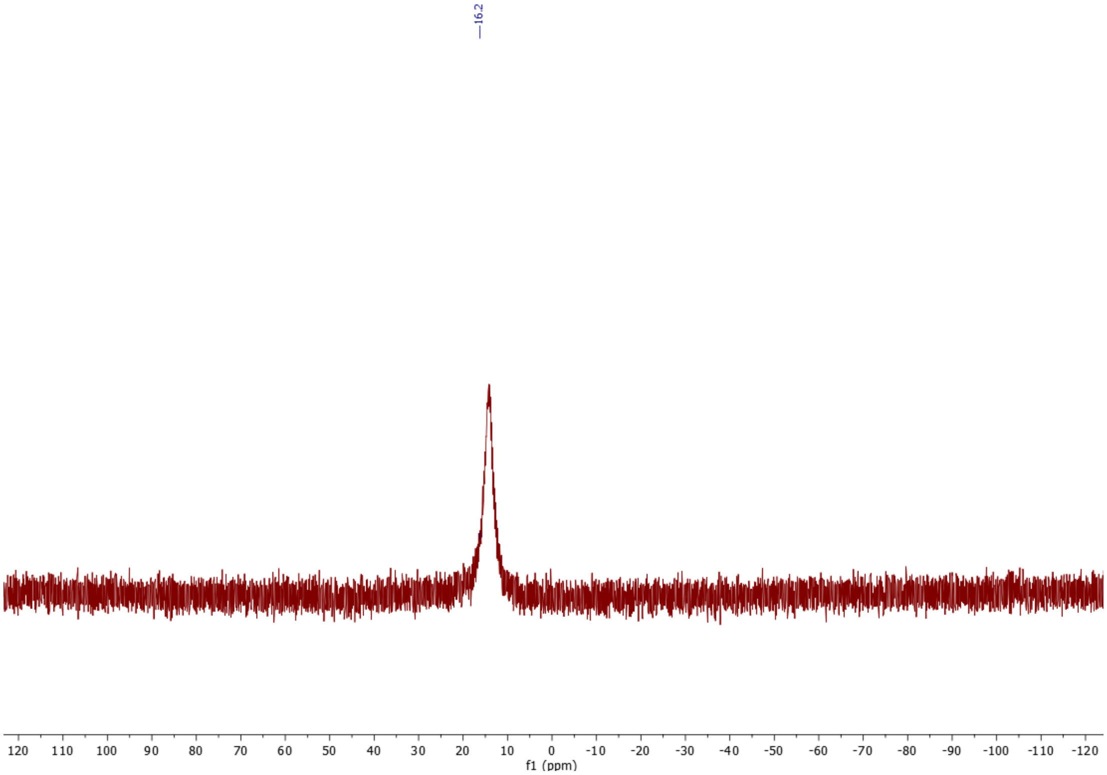

Supplemental References

1 Wiegand, I., Hilpert, K. & Hancock, R. E. W. Agar and broth dilution methods to determine the minimal inhibitory concentration (MIC) of antimicrobial substances. *Nature Protocols* **3**, 163-175, doi:10.1038/nprot.2007.521 (2008).

2 López, A., Clark, T. B., Parra, A. & Tortosa, M. Copper-Catalyzed Enantioselective Synthesis of β-Boron β-Amino Esters. *Organic Letters* **19**, 6272-6275, doi:10.1021/acs.orglett.7b02784 (2017).
