# Supplementary material for: A small molecule inhibitor prevents gut bacterial genotoxin production
Source: Nat Chem Biol. 2022 Oct 17;19(2):159–67. doi: 10.1038/s41589-022-01147-8 (PMC9889270; doi:10.1038/s41589-022-01147-8)
Supplement: Supplementary file 2 — Reporting Summary [file 41589_2022_1147_MOESM2_ESM.pdf]

nature research

Corresponding author(s): Balskus, Emily P.

Last updated by author(s): Aug 3, 2022

Reporting Summary

Nature Research wishes to improve the reproducibility of the work that we publish. This form provides structure for consistency and transparency in reporting. For further information on Nature Research policies, see our Editorial Policies and the Editorial Policy Checklist.

Statistics

For all statistical analyses, confirm that the following items are present in the figure legend, table legend, main text, or Methods section.

n/a	Confirmed	
☐	☒	The exact sample size (*n*) for each experimental group/condition, given as a discrete number and unit of measurement
☐	☒	A statement on whether measurements were taken from distinct samples or whether the same sample was measured repeatedly
☐	☒	The statistical test(s) used AND whether they are one- or two-sided<br>*Only common tests should be described solely by name; describe more complex techniques in the Methods section.*
☒	☐	A description of all covariates tested
☐	☒	A description of any assumptions or corrections, such as tests of normality and adjustment for multiple comparisons
☐	☒	A full description of the statistical parameters including central tendency (e.g. means) or other basic estimates (e.g. regression coefficient) AND variation (e.g. standard deviation) or associated estimates of uncertainty (e.g. confidence intervals)
☐	☒	For null hypothesis testing, the test statistic (e.g. *F*, *t*, *r*) with confidence intervals, effect sizes, degrees of freedom and *P* value noted<br>*Give P values as exact values whenever suitable.*
☒	☐	For Bayesian analysis, information on the choice of priors and Markov chain Monte Carlo settings
☒	☐	For hierarchical and complex designs, identification of the appropriate level for tests and full reporting of outcomes
☒	☐	Estimates of effect sizes (e.g. Cohen's *d*, Pearson's *r*), indicating how they were calculated

Our web collection on statistics for biologists contains articles on many of the points above.

Software and code

Policy information about availability of computer code

Data collection	Fluorescence and absorbance data was collected using Gen5 (BioTek) or Softmax Pro 7.0 (Molecular Devices); Mass Spectrometry data was collected using either MassLynx 4.2 (Waters), Mass Hunter B10 (Agilent) or the Orbitrap Lumos Instrument Control Software; Gels for activity based protein profiling were imaged using the GelOdyssey Imaging System; Western blots were imaged using an the Azure Sapphire c300 software; Flow cytometry data was collected using BD FACSDiva platform.
Data analysis	Quantitative mass spectrometric analysis was conducted using TargetLynx 4.2 (Waters). Other mass spectrometric analysis was conducted in Qualitative Analysis (Agilent), and Xcalibur (ThermoFisher Scientific). Metabolomic analysis was performed in MzMine 2.53 using the ADAP chromatogram builder algorithm. Flow cytometry analysis was conducted using FlowJo 10.7.1. Crystallography: XDS 0.86, AIMLESS 7.0.077, PHENIX 1.19.1-4122, COOT 0.9.4, DIALS 1.14.5-g19190e3b9-release; Additional data analysis was conducted in Microsoft Excel 2016 and GraphPad Prism 9.

For manuscripts utilizing custom algorithms or software that are central to the research but not yet described in published literature, software must be made available to editors and reviewers. We strongly encourage code deposition in a community repository (e.g. GitHub). See the Nature Research guidelines for submitting code & software for further information.

Data

Policy information about availability of data

All manuscripts must include a data availability statement. This statement should provide the following information, where applicable:

- Accession codes, unique identifiers, or web links for publicly available datasets
- A list of figures that have associated raw data
- A description of any restrictions on data availability

Atomic coordinates and structure factors for the reported crystal structures in this work have been deposited to the Protein Data Bank under accession number

7MDC. Corresponding X-ray diffraction images have been deposited to the SBGrid Data Bank under accession number 832 (doi:10.15785/SBGRID/832). All other data reported in this study can be obtained from the corresponding author upon request.

Field-specific reporting

Please select the one below that is the best fit for your research. If you are not sure, read the appropriate sections before making your selection.

- [x] Life sciences
- [ ] Behavioural & social sciences
- [ ] Ecological, evolutionary & environmental sciences

For a reference copy of the document with all sections, see nature.com/documents/nr-reporting-summary-flat.pdf

Life sciences study design

All studies must disclose on these points even when the disclosure is negative.

Sample size	No statistical methods were used to determine sample sizes in advance. All experiments were conducted in 3-5 biological replicates (see figure captions). For most direct measurements of inhibitor activity, a minimum sample size of 3 was chosen since preliminary experiments showed that these assays were robust and that effect sizes were large enough to distinguish statistically significant differences even at small replicate numbers. For assays with a larger n, additional replicates were added when possible to ensure robustness of results.
Data exclusions	No data were excluded from analysis.
Replication	All experiments here were conducted in at least 3 biological replicates and, in some cases, were repeated on different days following similar protocols. In all cases, the observations reported here were reproducible.
Randomization	This is not relevant to this study. All living organisms tested were genetically identical and derived from the same stock for all sample groups.
Blinding	Investigators were not blinded, since in most cases sample preparation, data collection, and analysis were all conducted by the same person. Every effort was made to rely on automated methods and algorithms to conduct measurements and data analysis in order to minimize the introduction of personal bias.

Reporting for specific materials, systems and methods

We require information from authors about some types of materials, experimental systems and methods used in many studies. Here, indicate whether each material, system or method listed is relevant to your study. If you are not sure if a list item applies to your research, read the appropriate section before selecting a response.

Materials & experimental systems

n/a	Involved in the study	
☐	☒	Antibodies
☐	☒	Eukaryotic cell lines
☒	☐	Palaeontology and archaeology
☒	☐	Animals and other organisms
☒	☐	Human research participants
☒	☐	Clinical data
☒	☐	Dual use research of concern

Methods

n/a	Involved in the study	
☒	☐	ChIP-seq
☐	☒	Flow cytometry
☒	☐	MRI-based neuroimaging

Antibodies

Antibodies used	mouse anti-actin (Thermo Fisher Scientific, Catalog # MA511869), mouse anti-FANCD2 (Thermo Fisher Scientific, Catalog # MA123347), peroxidase-conjugated goat anti-mouse (Jackson ImmunoResearch product 115-035-003).
Validation	All antibodies were validated by the manufacturer and used according to the manufacturer's recommended protocols. According to the manufacturer's (ThermoFisher/Invitrogen) website "This Antibody was verified by Knockout to ensure that the antibody binds to the antigen stated."

Eukaryotic cell lines

Policy information about cell lines

Cell line source(s)	American Type Culture Collection (ATCC) - HeLa (CCL-2)
Authentication	Cell lines were not authenticated

Mycoplasma contamination	Cell lines were not tested for mycoplasma.
Commonly misidentified lines (See ICLAC register)	None

Flow Cytometry

Plots

Confirm that:

- [x] The axis labels state the marker and fluorochrome used (e.g. CD4-FITC).
- [x] The axis scales are clearly visible. Include numbers along axes only for bottom left plot of group (a 'group' is an analysis of identical markers).
- [x] All plots are contour plots with outliers or pseudocolor plots.
- [x] A numerical value for number of cells or percentage (with statistics) is provided.

Methodology

Sample preparation	Cell lines were cultured and treated as described in the Methods. 24 hours after treatment, cells were released from plates by trypsinization, isolated by centrifugation, washed with phosphate buffered saline (PBS), and then fixed in cold 70% ethanol. Fixed cells were stored at 4 degrees centigrade for 24 hours before analysis. Cells were again isolated, then resuspended in PBS and treated with RNAse and propidium iodide (PI) to stain for DNA content.
Instrument	BD LSRII (Harvard University Bauer Core Flow Cytometry Facility)
Software	Data was collected using the BD FACSDiva package and analyzed in FloJo 10.7.1.
Cell population abundance	Samples were gated (see below) to remove debris and doublet cells/clusters from analysis. The remaining single cells were then plotted as histograms to show the distribution of cells based on DNA content (Figure 5). The Watson Model for cell cycle analysis was applied to each distribution distribution in order to determine the percentage of the population in G1 phase.
Gating strategy	After collecting at least 10,000 events for each sample, each sample was gated on a plot of FSC-A vs SSC-A to separate debris from cells (retaining 70-90% of events). This cell population was then gated on SSC-W vs SSC-A to separate single cells (91-98%). The single cell population was then gated on FSC-A vs propidium iodide fluorescence to remove unstained cells and other outliers (89-97%). This final population was then plotted as a histogram and fit to the Watson Model. In some cases, noise in the data made automatic unconstrained fitting impossible, the fitting process was aided by constraining the G1 peak center to the left (less fluorescent) half of the population and setting the condition that the G2 peak CV = G1 peak CV. In all cases, the same gate values were applied to every sample at each stage of gating.

- [x] Tick this box to confirm that a figure exemplifying the gating strategy is provided in the Supplementary Information.
