# Supplementary material for: A small molecule inhibitor prevents gut bacterial genotoxin production
Source: Nat Chem Biol. 2022 Oct 17;19(2):159–67. doi: 10.1038/s41589-022-01147-8 (PMC9889270; doi:10.1038/s41589-022-01147-8)

Replicate 1 – full chemiluminescence image

NOTE: in all images, the blot membrane is cut in half because the high molecular weight section had to be stained with a much higher concentration of the secondary antibody to be visible. No part of the membrane was removed.

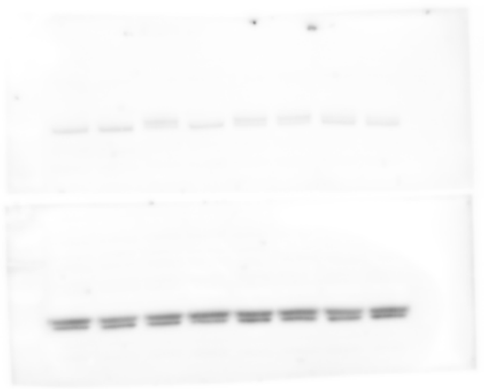

Replicate 1 – visible light marker image (for ladder)

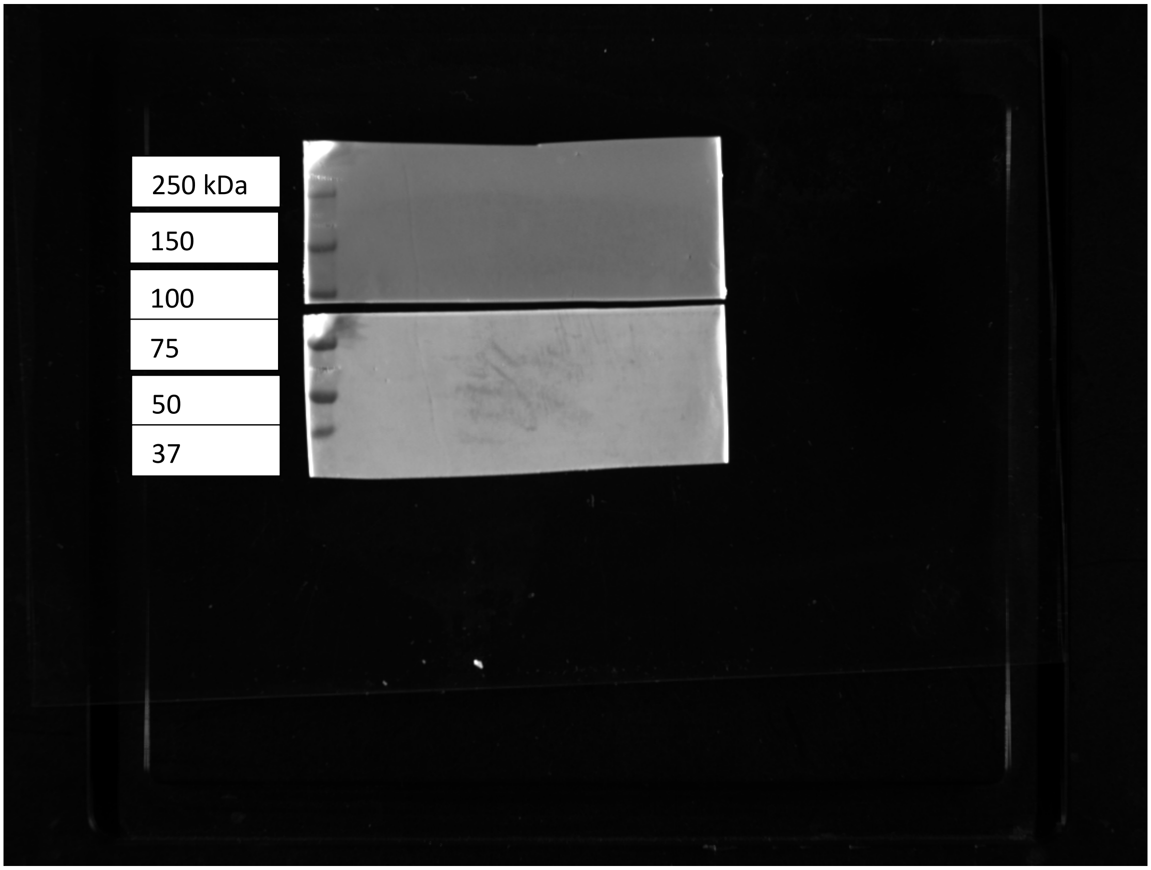

Replicate 2 – Chemiluminescence image

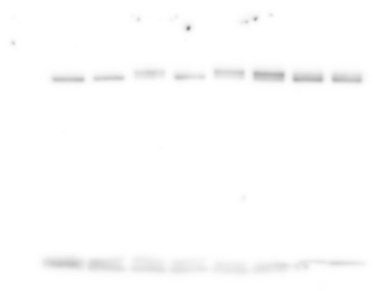

Replicate 2 – Visible light image

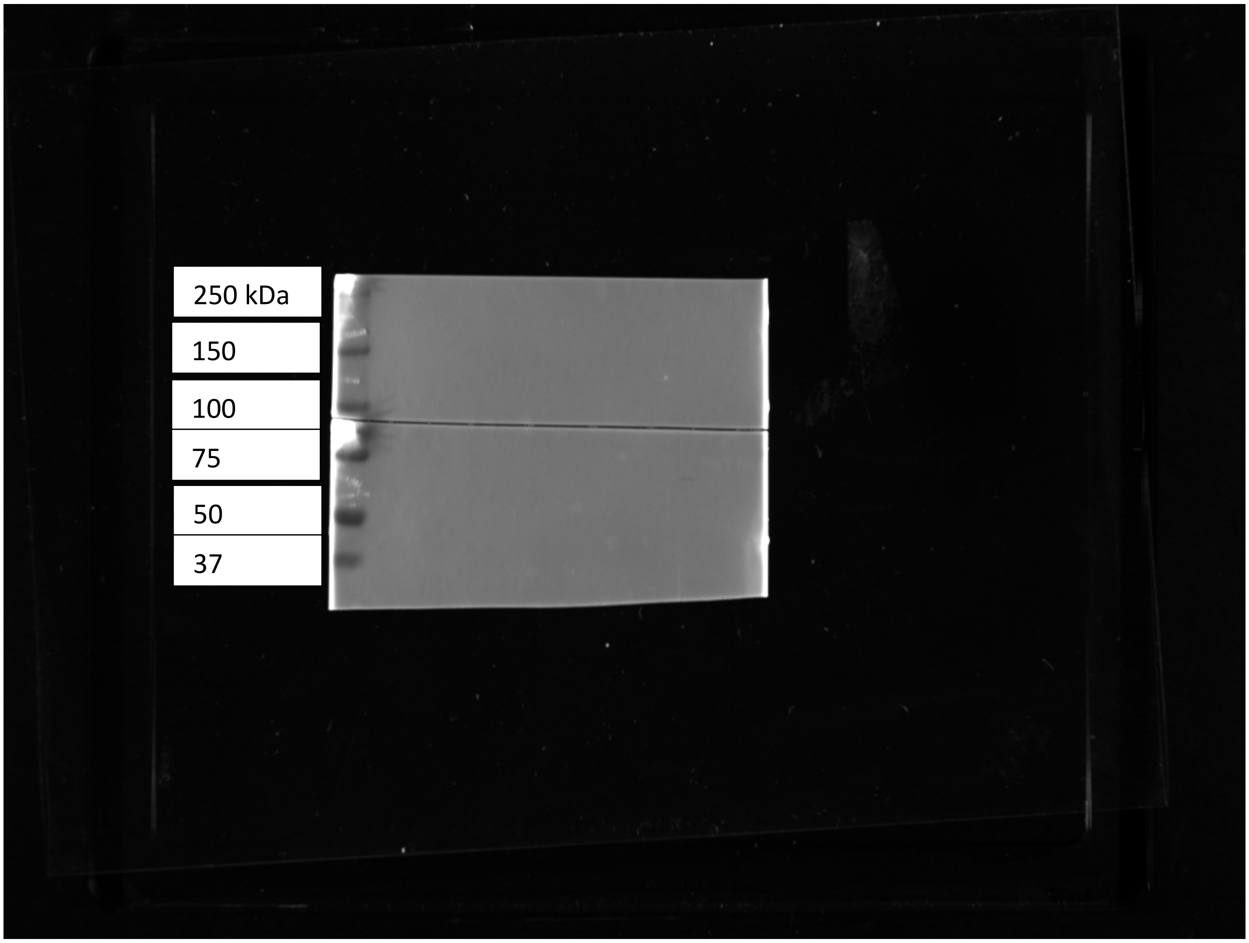

Replicate 3 – chemiluminescence image:

For this replicate, the actin loading control appears inconsistent due to a user error (gel overrun)

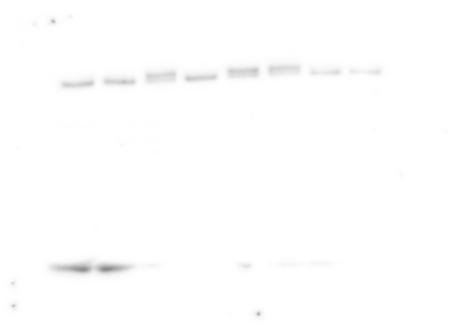

Replicate 3 – Visible light image

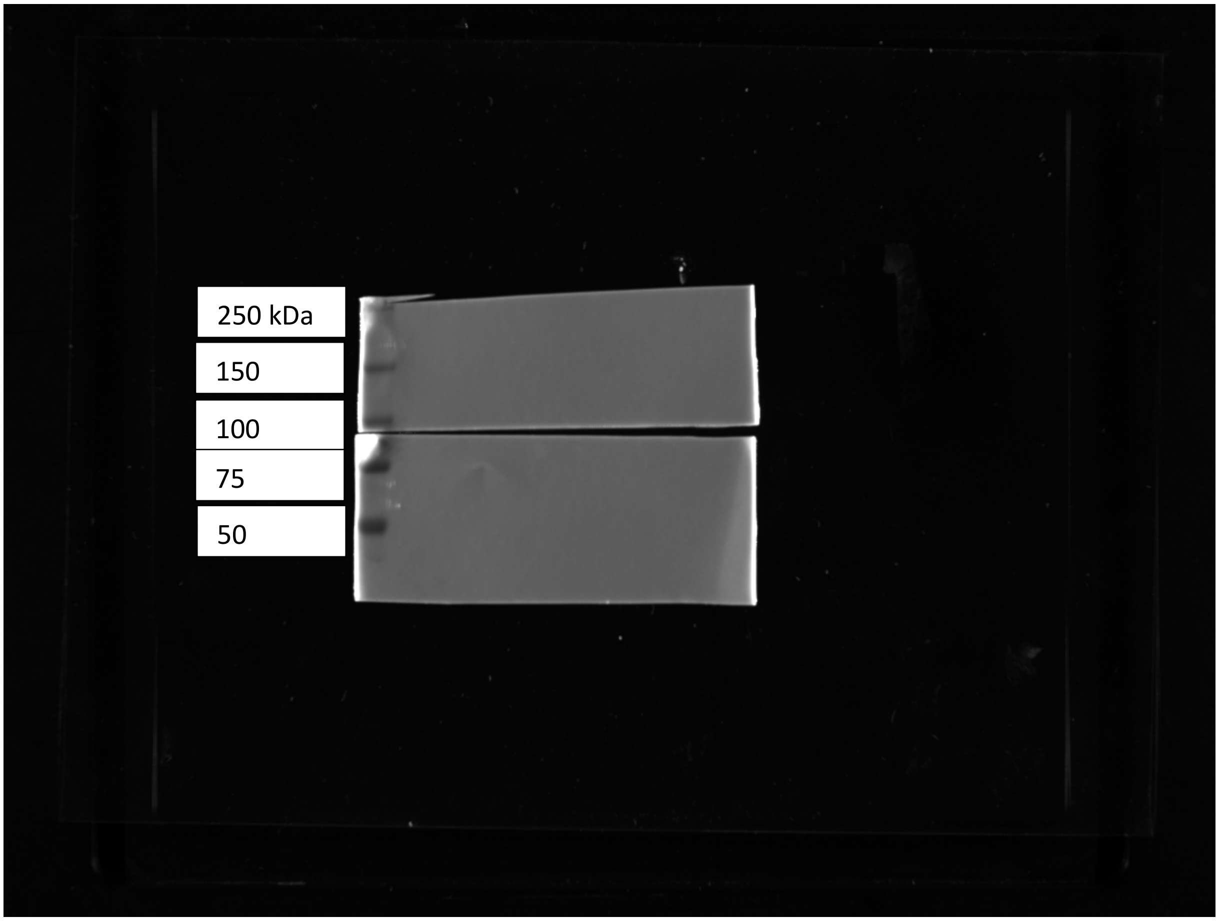

Supplement: Source Data Fig. 5 — Uncropped chemiluminescence and visible light images of Western blots for FANCD2. Additional Source Data for panels in Fig. 5 available online. See Data Availability Statement. [file 41589_2022_1147_MOESM5_ESM.pdf]
